# The Structural Difference of Isobaric *N*-Glycans of Two Microalgae Samples Reveals Taxonomic Distance

**DOI:** 10.3389/fpls.2021.643249

**Published:** 2021-04-26

**Authors:** Réka Mócsai, Hanspeter Kaehlig, Markus Blaukopf, Johannes Stadlmann, Paul Kosma, Friedrich Altmann

**Affiliations:** ^1^Department of Chemistry, University of Natural Resources and Life Sciences, Vienna, Austria; ^2^Department of Organic Chemistry, Faculty of Chemistry, University of Vienna, Vienna, Austria

**Keywords:** microalgae, *Chlorella*, *N*-glycan, arabinose, *Scenedesmus*

## Abstract

Microalgae of the *Chlorella* clade are extensively investigated as an environmentally friendly source of renewable biofuels and high-value nutrients. In addition, essentially unprocessed *Chlorella* serves as wholesome food additive. A recent study on 80 commercial *Chlorella* preparations revealed an unexpected variety of protein-linked *N*-glycan patterns with unprecedented structural features, such as the occurrence of arabinose. Two groups of products exhibited a characteristic major *N*-glycan isobaric to the Man_2_GlcNAc_2_XylFuc *N*-glycan known from pineapple stem bromelain, but tandem mass spectrometry (MS/MS) analysis pointed at two types of *N*-glycan different from the bromelain structure, as well as from each other. Here we report the exact structures of these two novel *N*-glycan structures, elucidated by nuclear magnetic resonance spectroscopy and MS/MS, as well as on their phylogenetic context. Despite their humble size, these two *N*-glycans exhibited a very different design with structural features unrelated to those recently described for other *Chlorella*-clade strains. The major glycans of this study presented several novel structural features such as substitution by arabinose or xylose of the internal *N*-acetylglucosamine, as well as methylated sugars. ITS1-5.8S-ITS2 rDNA barcode analyses revealed that the xylose-containing structure derived from a product primarily comprising *Scenedesmus* species, and the arabinose-containing glycan type related to *Chlorella* species (SAG211-34 and FACHB-31) and to *Auxenochlorella*. This is another example where characteristic *N*-glycan structures distinguish phylogenetically different groups of microalgae.

## Introduction

Microalgae of the genus *Chlorella* find a plethora of current and potential uses. Many hopes are lain in their ability to convert sewage into useful materials (Liu and Chen, 2016; Markou et al., 2018), to constitute a source of renewable fuel (Wang et al., 2020) or—at lower volumes but higher value—of a nonsynthetic source of chemicals, not at least food or feed additives such as polyunsaturated fatty acids (Haslam et al., 2020; Madhubalaji et al., 2020), carotenoids (Sun et al., 2019; Zhang et al., 2020), or many other pharmaceutically valuable compounds (Rosales-Mendoza et al., 2020). *Chlorella* is regarded as a potential future source of protein for direct consumption, as well as use in livestock farming (Amorim et al., 2020). Currently, a multitude of *Chlorella* tablets and powders are offered for human consumption as something between healthy food and food supplement, where the protein and/or vitamin content or other health benefits are emphasized. Chlorellaceae are unicellular, nonflagellated, essentially spherical freshwater algae and as such very poor in optical features that would allow strain discrimination. Even though a number of species have been defined, but rather obvious from the frequent changes (De Clerck et al., 2013), classification of a given isolate causes trouble. An example is posed by the strain FACHB-31 that originally was seen as *Scenedesmus obliquus* (Xu et al., 2015) and as such as a member of the class of Chlorellaceae, which against cursory impression does not comprise the family of the Chlorellales, which is home to *Chlorella*. Later, the same strain was designated *Chlorella* and thus fell or falls in the class of Trebouxiophyceae (Wu et al., 2018), which fits well with recent DNA-based phylogeny (Mocsai et al., 2020a). An example of two different classes in the animal area is the classes of birds and mammals, where misclassifications are hard to imagine.

The recent discovery of a stunning diversity of the structures of protein-linked glycans, so-called *N*-glycans, suggests *N*-glycan patterns as obtained by matrix-assisted laser desorption ionization-time-of-flight mass spectrometry (MALDI-TOF MS) as a straightforward and precise taxonomic criterion (Mocsai et al., 2020a). Glycan patterns align with genetic markers but pose a much more clear-cut distinction than DNA homologies. Mass spectrometry yields the composition in terms of number of hexoses, *N*-acetylhexosamines, pentoses, and methyl groups, whereby the mass of a deoxyhexose is equivalent to the sum of a pentose and a methyl group—wherever the latter may be located. For mammalian *N*-glycans, in which neither pentoses nor methyl groups are found, a widely used abbreviation scheme names glycans, e.g., H5N4F1, where F stands for fucose. To distinguish from this system, we refer to an oligosaccharide with four hexoses, 2 *N*-acetylglucosamines (GlcNAc), one pentoses, and three methyl group as os4213 (Mocsai et al., 2020a). The survey on by now more than 100 commercial products revealed not only the existence of different glycan patterns. Chromatographic comparison, tandem MS (MS/MS), and constituent analysis revealed that many identical masses actually arose from different structures. A most striking example was the os3231 (1,320.4 Da) that obviously can occur in three different structures (Mocsai et al., 2020b). All of them were found to contain arabinose, a component so far not observed in *N*-glycans. Further examples are os4221 that occurred in the “Jos” and the “Kei” glyco group and os2221 from the hardly distinguishable groups “Raa” and “Now.” These show a dominating MALDI-MS peak at *m/z* = 1,049.3, which equals the long known Man2GlcNAc2XylFuc *N*-glycan from pineapple stem bromelain, also known as MUXF^3^ (Altmann, 2007). MS/MS clearly revealed these os2221 as differing from the plant *N*-glycan and from each other.

For this article, we examined the *N*-glycan structures of the dominating os2221 glycan in the “Raa” and “Now” glyco groups by various techniques including two-dimensional (2D) nuclear magnetic resonance (NMR) spectroscopy. Several commercial *Chlorella* products could be assigned to the “Raa” group, and three strain collection lines exhibited this pattern (SAG211-31, SAG211-34, FACHB-31). The “Now” glycan pattern was found in only one commercial sample, and this orphan status found its repercussion in the results of ITS1 and ITS2 rDNA barcoding.

## Materials and Methods

### Sources of Microalgae and Extraction of *N*-Glycans

Live strains were obtained and grown as recently described (Mocsai et al., 2020a,b). Commercial samples were purchased as described in Supplementary Table 1. *N*-glycans were isolated by a combination of pepsin digestion, cation exchange, and size exclusion chromatography and peptide:*N*-glycosidase digestion as described (Mocsai et al., 2020a). Reducing or sodium borohydride reduced *N*-glycan mixtures were fractionated by hydrophilic liquid interaction chromatography (HILIC) on a TSK-amide 80 column (Tosoh Bioscience GmbH, Griesheim, Germany) (Mocsai et al., 2020b).

### MS and Glycosidase Probing of Glycans

MALDI-TOF MS and MS/MS were performed with 2,5-dihydroxybenzoic acid as the matrix on an Autoflex instrument (Bruker, Bremen, Germany). For electrospray MS/MS, reduced glycans were subjected to porous graphitic carbon chromatography, and the eluate was analyzed with a maXis 4G Q-TOF MS (Bruker) in either positive or negative mode (Mocsai et al., 2020a).

For compositional analysis, samples were hydrolyzed for 4 h at 100°C with 2 M trifluoroacetic acid. Monosaccharides were reduced with NaBD_4_ and analyzed by gas chromatography (GC)–MS as alditol acetates (Mocsai et al., 2019, 2020b). For linkage analysis, permethylation of oligosaccharides was achieved with iodomethane and sodium hydroxide in dimethyl sulfoxide.

Individual fractions of “Now” C-5 were treated with α-mannosidase from jack beans (Sigma–Aldrich) in 100 mM MES buffer pH 6.3 at 37°C.

### Glycoproteomics

Algae tablets (approximately 500 mg) were suspended in 10 mL 6 M guanidium hydrochloride, 100 mM HEPES, pH 7.8, and incubated in a boiling water bath for 30 min. After cooling, 1 mL aliquots were diluted in 9 mL 100 mM HEPES, pH 7.8, and digested with 1 mg trypsin (TPCK-treated trypsin from bovine pancreas; Sigma), at 37°C, overnight. The tryptic digests were adjusted to pH 2 by dropwise addition of 10% trifluoroacetic acid and spun for 5 min at 10,000 × *g*. Tryptic (glyco-)peptides were isolated from these supernatants using C18 reversed-phase SPE cartridges (Chromabond C18ec, 1,000 mg) and dried in a SpeedVac concentrator. Glycopeptides were enriched by ion-pairing HILIC using SPE cartridges [Chromabond OH (Diol), 100 mg; Macherey-Nagel, D]. In brief, tryptic glycopeptides were resuspended in 1 mL 80% acetonitrile containing 1% trifluoroacetic acid and were loaded onto preconditioned OH SPE cartridges. The cartridges were then washed three times with 1 mL of 70% acetonitrile containing 1% trifluoroacetic acid, before eluting the glycopeptides in 500 μL of 50% acetonitrile in water.

Prior to liquid chromatography (LC)–electrospray ionization (ESI)–MS/MS analysis performed on a maXis 4G Q-TOF instrument (Bruker Daltonics; equipped with nano-ESI source) as described previously (Hennicke et al., 2017), the glycopeptides were dried in a SpeedVac concentrator and resuspended in 100 μL water. Additionally, 50 μL of each sample was adjusted to pH 5 by stepwise addition of 1 M sodium acetate, pH 5.0, and incubated with PNGase A at 37°C overnight.

Manual data interpretation and *de novo* sequencing of glycopeptides were performed using the vendor-specific data-analysis software package Data Analyst (Bruker Daltonics, Bremen, Germany).

### NMR Spectroscopy

The NMR spectra were recorded on a Bruker AV III HD 700 MHz NMR spectrometer (Bruker BioSpin, Rheinstetten, Germany). The instrument is equipped with a quadruple (^1^H, ^13^C, ^15^N, ^19^F) inverse helium cooled cryo probe operating at 700.40 MHz for ^1^H, and 176.12 MHz for ^13^C, respectively.

The spectra, all acquired at a temperature of 25°C in D_2_O as solvent, were referenced for ^1^H to the signal of the methyl groups of DSS (δ = 0 ppm). Chemical shifts for ^13^C are reported on a unified scale relative to ^1^H using the Ξ value for DSS (Harris et al., 2008).

For all 1D and 2D NMR experiments, the appropriate pulse sequences were used as supplied by the manufacturer. Regarding 1D spectra, ^1^H NMR was done with and without suppression of the HDO signal using presaturation; for ^13^C spectra, a DEPTq sequence with a 135° pulse for multiplicity selection was used. The following 2D experiments were performed: double quantum filtered (DQF) COSY, TOCSY (100-ms MLEV17 spin-lock), NOESY (800-ms mixing time), ROESY (250-ms spin-lock), HSQC with and without ^13^C decoupling, and HMBC. In order to get high-resolution spectra of the individual sugar spin systems, 1D selective TOCSY experiments were performed using a selective pulsed field gradient spin echo sequence with a 80-ms 180° Gaussian pulse for excitation of the appropriate signals, mainly the anomeric protons, and a MLEV17 spin-lock in the range from 100 to 300 ms, depending on the size of the spin–spin coupling.

The analysis of the spectra was done within the TopSpin software (Bruker BioSpin). To elucidate the spin coupling network of the individual sugars, spin simulations were done using DAISY within the TopSpin software, which is based on solving the time-independent Schroedinger equation to calculate the energy levels.

### DNA Extraction and DNA Barcode Analysis

Total genomic DNA was extracted from 30 mg dry algal powder using QIAGEN DNeasy^®^ Plant Mini Kit (Qiagen, Hilden, Germany) according to the producer’s instructions. The ITS1-5.8S-ITS2 DNA fragment was amplified by polymerase chain reaction (PCR) reaction with primers binding to the flanking regions of 18S and 26S rRNA gene, resulting in a DNA fragment of approximately 950 bp. The sequences of the primers were as follows: 5′→3′ TGCCTAGTAAGCGCAAGTCA (forward) and 5′→3′ TTCCTCCGCTTATTGATATGC (reverse). All PCR reactions were performed with the AccuTaq LA DNA polymerase (Sigma–Aldrich, St. Louis, MO, United States) according to the manufacturer’s instructions in a total volume of 20 μL using 1.5% dimethyl sulfoxide. After agarose gel electrophoresis, the PCR products were purified using the Illustra GFX PCR DNA and Gel Band Purification Kit (GE Healthcare, Vienna, Austria). The purified PCR product of the sample “Raa” C-76 was directly sent for Sanger sequencing—service provided by Microsynth AG (Balgach, Switzerland). For the “Now” sample, the PCR fragment was ligated into PminiT plasmids and transformed into 10-beta cells using the NEB PCR cloning kit (New England Biolabs, Frankfurt am Main, Germany) according to the manufacturer’s instructions. Seventeen single colonies were picked and cultured overnight in 5 mL Luria–Bertani/ampicillin medium at 37°C, and their plasmid DNA was extracted using a NucleoSpin Plasmid Kit (Thermo Fisher Scientific) following the manufacturer’s recommendations. Plasmid DNA was sent for Sanger sequencing. Sequencing results obtained from the respective forward and reverse primer pairs were aligned, generating nucleotide sequences that can be found in the Supplementary Data.

### Phylogenetic Analysis

All acquired ITS1-5.8S-ITS2 nucleotide sequences were aligned together with reference sequences from GenBank annotated as microalgae species, using the MAFFT algorithm L-INS-i (Katoh and Standley, 2013). A maximum likelihood phylogenetic tree was produced using PhyML software (Guindon et al., 2010) at the web server NGPhylogeny.fr (Lemoine et al., 2019) using SPR moves to optimize tree topology and five random starting trees. Statistical branch support was calculated using 500 bootstrap replications, and the optimal substitution model was assessed by Smart Model Selection (Lefort et al., 2017) to be GTR + G + I + F under the Akaike information criterion. Multiple sequence alignments and phylogenetic trees were visualized in the Jalview sequence analysis tool (Waterhouse et al., 2009) and with the MEGA7 software (Kumar et al., 2016), respectively. A comparable topology was achieved using ClustalOmega (Madeira et al., 2019) for sequence alignment (data not shown).

## Results

### Composition of the Major Peak in Microalgae With a FACHB-31–Like *N*-Glycan Pattern

**FIGURE 1:**
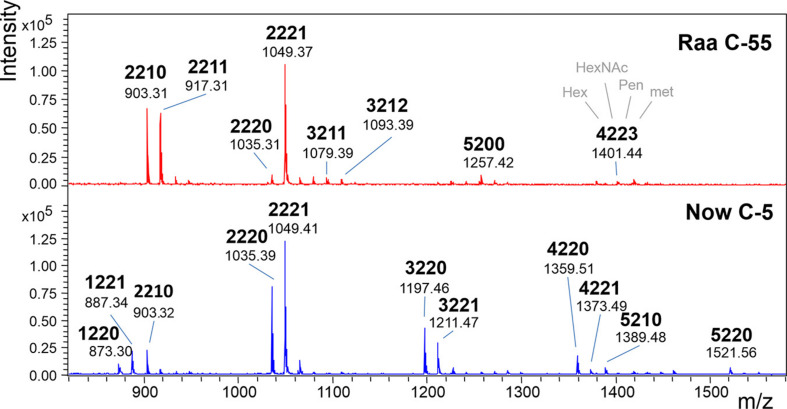
MALDI-TOF MS spectra of “Raa” and “Now” type total *N*-glycans. The 4-digit code interprets the [M+Na]^+^ ions in terms of numbers of hexoses, *N*-acetylhexosamines, pentoses, and methyl groups. Complementary mass spectra are deposited in Supplementary Figure 1.

The MALDI-TOF MS spectra of a number of commercial samples and type collection strains (i.e., the often-used strains FACHB-31 and SAG211-34) were highly similar with one dominating peak at *m/z* = 1,049.3 [M+Na]^+^ (Table 1). This mass is known from land plants, where it derives from a glycan with two *N*-acetyl glucosamines, two mannoses, a xylose, and a fucose, the so-called MUXF^3^ structure (Altmann, 2007). However, further satellite peaks at 903.3, 1,225.3, and 1,339.3 pointed at the possible presence of methyl groups (Figure 1). In one sample (“Now” C-5), the accompanying peak “flora” was more pronounced, and peaks at *m/z* = 1,035.3 and 1,197.3 clearly indicated the presence of two pentoses rather than a pentose and a deoxyhexose. Another distinctive feature of this sample was the strong *O*-methylation of oligomannosidic glycans up to Man9 (Supplementary Figure 1; Mocsai et al., 2020b). This unparalleled profile was termed “Now” glycan pattern, whereas the more frequently occurring profile got the name “Raa”. The major peak of *m/z* = 1,049.3 of the “Now” sample and one example of the “Raa” group (C-76) were isolated by HILIC and subjected to gas chromatographic monosaccharide analysis that unequivocally confirmed the founding of two different glycan groups (Supplementary Figure 7 of Mocsai et al., 2020a). “Now” contained two xylose residues, one *O*-methylated at position 3, whereas the “Raa” sample contained two arabinoses and a 3-*O*-methylated mannose as already reported in Mocsai et al. (2020a).

**TABLE 1:** List of microalgae products for which a dominating peak at m/z = 1,049.365 (= calculated mass) in MALDI-TOF MS was seen.

**Vendor/strain number^1)^**	**Major peak [M+Na]^+^**	**Interpretation (H N P me)**	**Composition of major peak**		**Some typical minor peaks**		**GenBank #**
	***m/z***	***mol/mol***	**Constituents**	***mol/mol***	***m/z***	***mol/mol***	
**“Raa” glyco group**							
55	1,049.37	2 2 2 1			771.26	2 2 0 0	
					903.31	2 2 1 0	
					917.33	2 2 1 1	
					1,079.39	3 2 1 1	
					1,093.39	3 2 1 2	
64	1,049.39	2 2 2 1			”	”	
76	1,049.40	2 2 2 1	Man	1	”	”	
			3-*O*-me-Man	1			
			Ara	2			
			GlcNAc	2			
6, 60, 63, 68, 70, 95, 96, 98, 102, 110	1,049.40	2 2 2 1			”	”	
FACHB-31	1,049.30	2 2 2 1			”	”	MK248017
SAG211-34	1,049.38	2 2 2 1			”	”	MN194596
**“Now” glyco group**							
5	1,049.41	2 2 2 1	Man	2	837.30	1 2 2 0	See^2)^
			Xyl	1	887.34	1 2 2 1	
			3-*O*-me-Xyl	1	903.32	2 2 1 0	
			GlcNAc	2	1,035.39	2 2 2 0	
					1,197.46	3 2 2 0	
					1,211.47	3 2 2 1	
					1,359.51	4 2 2 0	
					1,373.49	4 2 2 1	
					1,521.56	5 2 2 0	

*The grouping is based on the pattern of smaller peaks and on methylation of oligomannosidic glycans (Figure 1). The deduced composition is based on the monosaccharide analysis reported previously (Mocsai et al., 2020a).*

*1) Explicit details are listed in the Supplementary Information.*

*2) ITS1-5.8S-ITS2 sequence of Now C-5: only one example is given below. Sequences of other, highly homologous clones found in this product are listed in the Supplementary Information.*

*ACACACCGCCCGTCGCTCCTACCGATTGGGTGTGCTGGTGAAGTGTTCGGATTGGCAGCTTAGGGTGGCAACACCTCAGGTCTGCCGAGAAGTTCATTAAACCCTCCCACCTAGAGGAAGGAGAAGTCGTAACAAGGTT TCCGTAGGTGAACCTGCGGAAGAATCATTGAATTATTAAAACCACAATGTGAACCTTATTGTTCCGTGCCTTTGCTGCCGGCAAGGCAATCAGCTTTGCCTGATTGTACTTGCAAGCTGGTGCGAGTTTTATACTTGCATCAG TGGCGCTCTGGCATGCTTATACACCAGTGCTAACCACTGTCAAAACCAAACTCTGAAGCTTTGATTGCTATTAATTGGCAATCTTAACCAAAGACAACTCTCAACAACGGATATCTTGGCTCTCGCAACGATGAAGAACGCA GCGAAATGCGATACGTAGTGTGAATTGCAGAATTCCGTGAACCATCGAATCTTTGAACGCATATTGCGCTCGAGCCCTCGGGCAAGAGCATGTCTGCCTCAGCGTCGGTTTATAACCTCACCCCTCTCTCCTTTTGGAGAG CTGGTTAGCTTCTAGCTGGCCTTAGGAGTGGATCTGGCTTTCCCATTTGGTTTATTCTGAATGGGTTGGCTGAAGCTTAGAGGCTTAAGCAAGGACCCGATATGGGCTTCAACTGGATAGGTAGCACCGGCTTCTGCCGAC TACACGAAGTTGTGGCTTGTGGACTTTGCTAGAGGCCAAGCAGGAAACATGCTTTGCATGTCTTAAACTTTCGACCTGAGCTCAGGCAAGG.*

### Mass Spectrometric Characterization of “Raa” and “Now” Main Peaks

**FIGURE 2:**
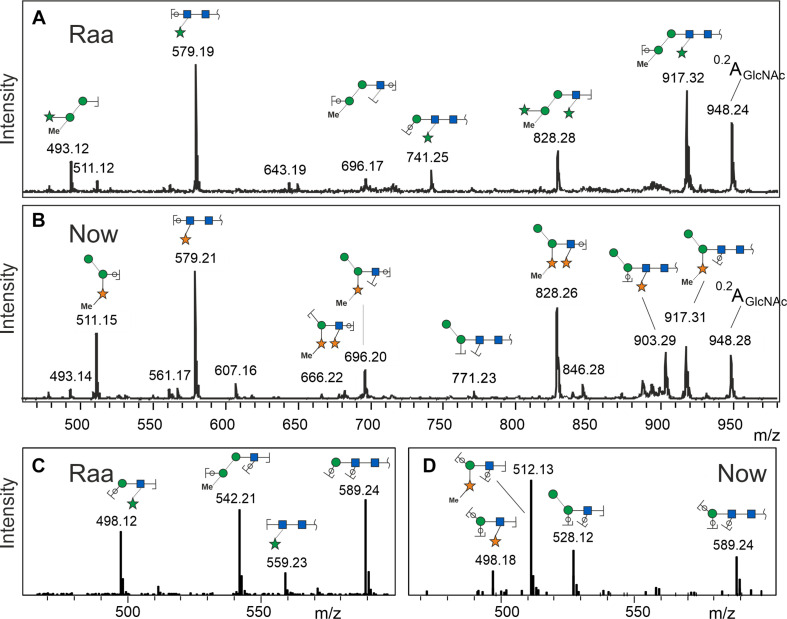
Fragment spectra of the major peak (os2221) by laser-induced dissociation in MALDI-TOF MS **(A,B)** and collision-induced dissociation in ESI-MS **(C,D)**. Singly charged Na^+^ or doubly charged, respectively.

MALDI-TOF MS/MS spectra at first sight looked rather similar with a strong fragment at 579.2, which can be explained as two GlcNAc and one pentose residue. The peak at *m/z* = 828.3 derived from loss of the reducing GlcNAc, which therefore could not have been substituted. From these two fragments, the valuable information arose that both glycans were substituted by a pentose on the second GlcNAc residue. The b- and c-type peaks at 493.2 and 511.2 comprised the other half of the glycans in both cases with a remarkable yet unexplained bias regarding b- and c-ions (Figure 2). Apart from this bias, the “Raa” and “Now” spectra looked very similar except from a rather small fragment at 903.3 that indicated loss of a methyl-pentose from 1,049.3 in “Now”.

Negative mode MS/MS as well as MALDI-TOF- or ESI-MS/MS of permethylated glycans failed to disclose any difference of the two samples. In the previous work, however, positive mode ESI-MS/MS had yielded clearly different fragmentation patterns, where fragment 498.2 occurred in both glycans. Fragment 512.2, however, turned up only in the “Now” sample. This indicated a sequence of me-Xyl-Man-GlcNAc. Given the inclination for gas-phase rearrangements of proton adduct ions (Wuhrer et al., 2009), we sought independent evidence for this interpretation. This came at no cost from a look at the MALDI-TOF MS spectrum where peaks with m/z = 873.3 and 887.3 can be seen in the “Now” spectrum (Figure 1). These peaks stand for glycans with two pentoses but only one hexose.

### Linkage Analysis by GC-MS

Linkage analysis of os2221 in “Raa” C-76 showed two mannose peaks, one with a substitution at position 3 and the other at position 4 (Table 2 and Supplementary Figure 2). Composition analysis had revealed a 3-*O*-methylated mannose, which presumably was an α1,3-mannose that also carried the arabinose in 4-position. The β-mannose would then have a free 6-position. This is a very unusual finding that, however, was confirmed by the NMR analysis (see below). A second arabinofuranose was linked in α1,3 position to the second GlcNAc residue, another unusual finding.

**TABLE 2:** Results of linkage analysis via permethylation and GC-MS analysis of partially methylated alditol acetates of the isolated major peaks (os2221) of two microalgae products.

**Partially methylated alditol acetates derived from:**	**Retention time (min)**	**“Raa” C-76**	**“Now” C-5**
		
		**Estimate of molar content (mol/mol)**	
Terminal arabinose	8.82	2	—
Terminal xylose	9.63	—	1
Terminal 3-*O*-methyl xylose	9.63	—	1
Terminal mannose	13.44	—	1
4-substituted 3-*O*-methyl mannose	16.87	1	—
3-substituted mannose	17.16	1	—
2,6-di-substituted mannose	21.19	—	1
4-substituted GlcNAc	27.41	1	1
3,4-di-substituted GlcNAc	30.70	1	1

In linkage analysis with deuteromethyl iodide, the “Now” C-5 major *N*-glycan gave a large peak for terminal xylopyranose, which in part carried a natural methyl residue. Besides a terminal mannose, a 2,6-substituted mannose reminded of the situation pineapple stem bromelain with its xylose in 2-linked position (Table 2). The second xylopyranose was linked in β1,3 position to the second GlcNAc residue.

“Now” C-5 contained a series of glycans with two pentoses and one to five hexoses. Fractions containing os5220 and os3220 were probed with jack bean α-mannosidase. The results indicated that all hexose residues are mannoses (Supplementary Figure 3).

### NMR Analysis of the Major *N*-Glycans

**FIGURE 3:**
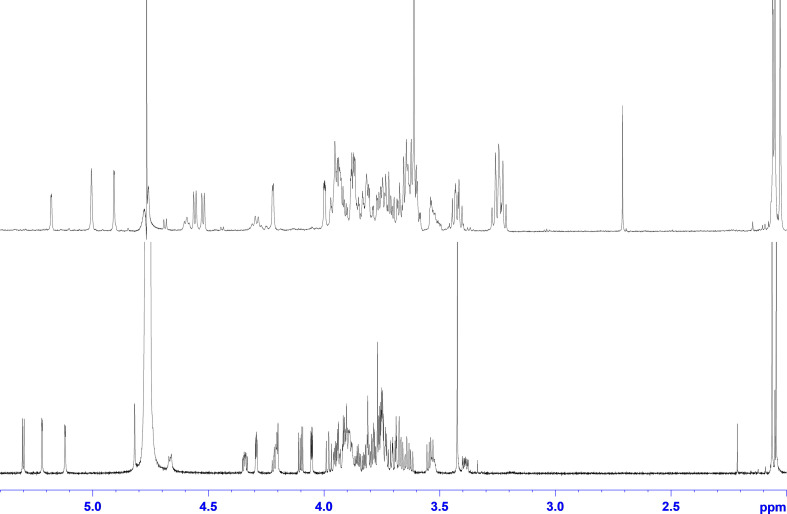
^1^H NMR spectra of glycan preparations: bottom trace, “Raa”; top trace, “Now.”

Two individual glycan preparations with identical monoisotopic mass of m/z = 1,026.3, “Raa” C-76 and “Now” C-5 sample, were subjected to a detailed NMR analysis. Figure 3 shows the ^1^H NMR spectra of “Raa” together with “Now” on top. Although the two spectra look significantly different, common features for both are a well-resolved anomeric region from 4.5 to 5.3 ppm, two acetyl signals at 2 ppm as well as a signal for a methoxy group at 3.4 or 3.6 ppm. Looking closer to the anomeric region, only five signals can be seen for “Raa” as this sample is derived from a sodium borohydride reduced *N*-glycan and thus terminated with an alditol. On the other hand, “Now” has a free reducing end, thus giving an anomeric mixture with seven signals in this region. HSQC spectra show the corresponding anomeric carbons in a spectral range from 93 to 110 ppm (Supplementary Figures 4, 5). The assignment of all proton and carbon signals was achieved using DQF-COSY, TOCSY, NOESY or ROESY, HSQC, and HMBC experiments. To overcome the low resolution of the 2D spectra and to identify the protons in the very overcrowded core region between 3.2 and 4.3 ppm, 1D TOCSY experiments were performed starting from the well-resolved anomeric signals. As a result, the entire spin system for all sugars can be made visible in high resolution (Supplementary Figures 6, 7). The extracted chemical shifts and spin couplings could be verified for all sugars by a spin simulation. All NMR data are summarized in the Supplementary Material (Supplementary Tables 2, 3).

Starting with the “Raa” preparation, the two downfield anomeric signals at 5.30 and 5.22 ppm gave spin systems in the TOCSY experiments, which can be addressed to pentoses. HMBC correlations from these protons to the corresponding carbon 4 at 83.9 or 86.6, respectively, established a furanose configuration for both. All proton and carbon chemical shift data together confirmed the result from the MS analysis for these two sugars being arabinoses. The anomeric configuration is β for the arabinofuranose with the more shielded anomeric proton and α for the other one, which can additionally be certified by appropriate NOESY crosspeaks. The next two anomeric signals going to higher field at 5.12 and 4.82 ppm have both only a very tiny splitting with proton 2. This small J coupling followed by another one of about 3 Hz between H2 and H3 identifies these sugars being of mannose type. The heteronuclear J coupling derived from a ^13^C-coupled HSQC spectrum was used for assignment of the anomeric configuration, which is α for the first one (^1^J_1H,13C_ = 172.5 Hz) and β for the second (^1^J_1H,13C_ = 161.2 Hz). The α-Man*p* is methylated at position 3 as can be seen from an HMBC crosspeak between the CH_3_ protons and carbon 3 of the mannose. The last remaining anomeric signal at 4.67 ppm is broadened with a large splitting of 7.8 Hz. The following spin couplings in the spin system again are large axial–axial interactions certifying a glucose-type sugar. Moreover, carbon 2 with a chemical shift of 58.1 ppm finally identifies this residue as β-Glc*p*NAc. The terminal residue at the reducing end is, as mentioned above due to the preparation an alditol, according to a chemical shift for C2 of 55.6 ppm derived from an amino sugar as well. This finding is a verification of an unsubstituted Glc*p*NAc at the reducing end proposed by the MS analysis.

**FIGURE 4:**
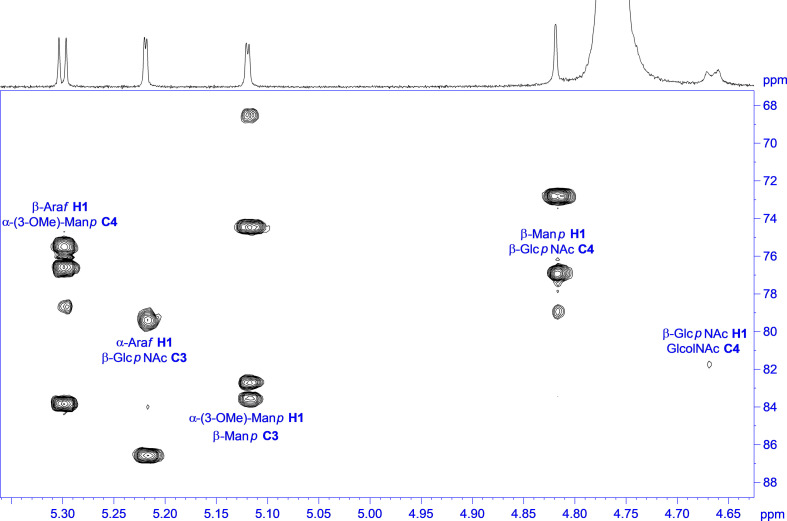
^1^H,^13^C HMBC spectrum of “Raa” with annotation for the linkage crosspeaks.

The linkage information between the building blocks for establishing the final glycan structure of “Raa” was derived unambiguously from HMBC correlations, namely, from the anomeric protons over the glycosidic bond to the corresponding carbons of the aglycon (Figure 4), starting from the alditol, connected to position 4 is β-Glc*p*NAc, which is linked likewise at position 4 to β-Man*p* and in addition is substituted by α-Ara*f* at position 3. Going on in sequence, the β-Man*p* is linked at position 3 to the α-3-(*O*-Me)-Man*p*, which has β-Ara*f* attached to position 4.

The analysis of the “Now” glycan was done in a similar way, hampered by a free reducing end, which doubles signals for the two anomeric forms not only for the terminal sugar, but also ongoing in sequence, and thereby reducing intensity for the individual lines as well. Because of their lower intensity, the anomeric protons for the reducing sugar can be identified at 4.69 ppm with a splitting of 8.2 Hz (β), and at 5.18 ppm with a splitting of only 2.5 Hz (α). As all other J couplings are in the range from 9 to 10 Hz, and carbons 2 have a chemical shift of 58.8 and 56.4 ppm, respectively, the terminal sugar at the reducing end is a Glc*p*NAc. Next two pentose spin systems can be found starting from the anomeric signals at 4.52 and 4.56 ppm; both can be addressed to β-xylopyranoses, as all J couplings are large axial–axial interactions. In addition, the second one is methylated, proven by an HMBC crosspeak between the methyl protons and C3 of the β-Xyl*p*. Then two anomeric signals at the down field side of the residual water signal at 4.91 and 5.00 ppm have only a very small J coupling to H2, and as the J coupling between H2 and H3 is small as well, both sugars are of mannose type. A heteronuclear ^1^H-^13^C spin coupling of 159.2 Hz provided β configuration for the first one, and 172.8 Hz α for the second. Finally, one anomeric signal is left at 4.59, which is again broadened similar as for the “Raa” glycan. The analysis of the spin system results in β-Glc*p*NAc for this sugar.

**FIGURE 5:**
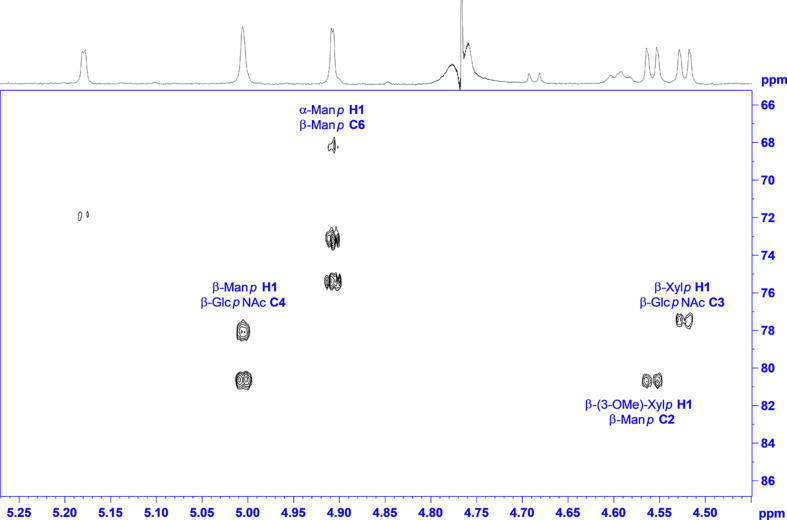
^1^H,^13^C HMBC spectrum of “Now” with annotation for the linkage crosspeaks.

The main source to establish linkage information is again an HMBC experiment. Unfortunately, no crosspeaks can be seen to the reducing sugar due to the low sensitivity. Instead, NOESY crosspeaks can be seen between H4 and the anomeric proton of the second amino sugar, and in addition, a chemical shift of about 82 ppm for C4 certifies this 1-4 linkage. Like in “Raa”, the β-Glc*p*NAc at the reducing end is unsubstituted. The next connectivities are accessible from HMBC correlations (Figure 5). The β-Glc*p*NAc number two in sequence is linked at position 4 to the β-Man*p* and is in addition decorated at position 3 with a β-Xyl*p*. Likewise, the β-Man*p* is substituted with the other methylated β-Xyl*p* at position 2. The terminal sugar at the nonreducing end is the α-Man*p*, which is 1-6 linked to the β-Man*p*.

**FIGURE 6:**
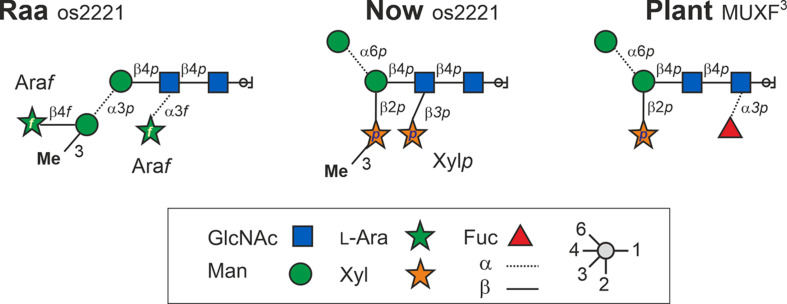
Structures of the major *N*-glycans found in sample C-76 (“Raa” type) and C-5 (“Now” type) both having the mass 1,026.3 Da. Os2221 indicates the oligosaccharides’ numbers of hexoses, HexNAc, pentoses, and methyl groups, respectively. Bromelain MUXF^3^ depicted for comparison.

Taken together and with independent support from mass spectrometric results, the structures of the major glycan species of the “Raa” and the “Now” glyco group are as shown in Figure 6 and Supplementary Figures 8, 9.

### Glycopeptide Identification

**FIGURE 7:**
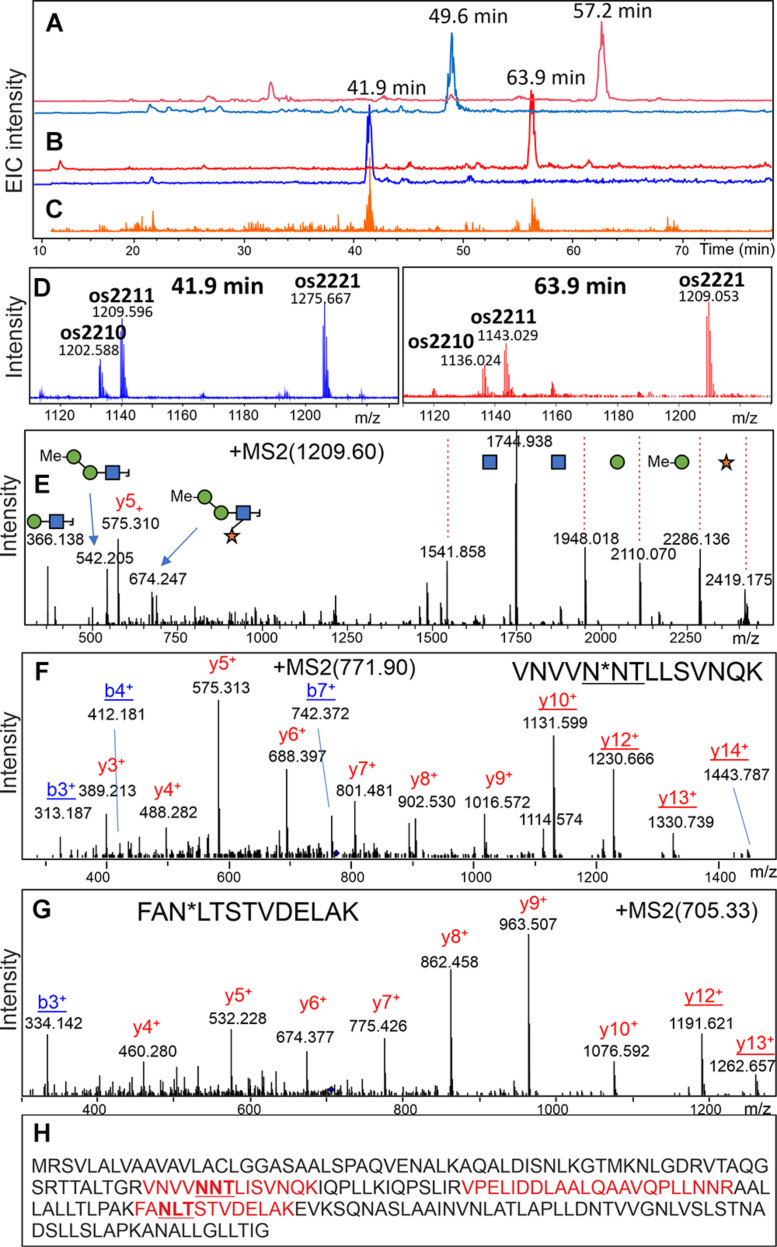
Discovery of a glycopeptide in a “Raa” glyco-type sample. A search for glycopeptides was conducted in a HILIC-enriched fraction of tryptic peptides of product “Raa” C-76 by using the XIC for m/z = 366.1 **(C)**. MS XICs for m/z = 1,276.12 and 1,209.57 Th are shown in panel **(B)** with the respective MS sum spectra in panel **(D)**. The pattern of glycoforms resembled that of the free glycans (Figure 1). The fragment spectrum for the peak with 1,209.59 Th confirms its glycopeptide nature **(E)**. Upon deglycosylation, the respective peptides could be found **(A)**, and their MSMS spectra allowed *de novo* sequencing **(F,G)**. A list of experimental and calculated masses is provided as Supplementary Table 3. Sequence of the hypothetical protein with found peptides highlighted in red **(H)**. The MS/MS spectrum of the nonglycopeptide is shown in Supplementary Figure 9.

To identify the major glycoprotein constituents of the *Chlorella* products, tryptic glycopeptides were enriched by HILIC-SPE and subsequently analyzed by LC-ESI-MS/MS. Based on the detection of glycan-specific oxonium ions in numerous MS/MS spectra (e.g., oxonium ion 366.14 Da), manual data interpretation led to the identification of two glycopeptides with complex-type *N*-glycans in “Raa” C-55 (Figure 7 and Supplementary Table 4). *De novo* sequencing of the corresponding deglycosylated peptides yielded the sequences VNVVND^∗^TiiSVNQK and FAD^∗^iTSTVDEiAK, where ^∗^ denotes the aspartate residue generated by deglycosylation, and “i” stands for either Ile or Leu (Figure 7). Both peptides perfectly aligned to a hypothetical protein predicted in the course of genome sequencing of a *Rhizobium* isolate from a Central European freshwater lake (BioSample: SAMN02925445; NCBI RefSeq: WP_054158787.1; Uniprot: A0A0N1KWP3). The nucleotide sequence of this gene aligned to another whole-genome sequence (ANZC01001603.1) that, however, originated from strain FACHB-9 (classified with the outdated species name *Chlorella pyrenoidosa*) (Fan et al., 2015). Although 100% identical, this sequence only covered the N-terminal part of the *bona fide* protein until LIRVPE. The last three residues find themselves in the nonglycosylated peptide VPELIDDLAALQAAVQPLLNNR (Figure 7, Supplementary Figure 10, and Supplementary Table 4), supporting the idea that both genomic sequences in fact describe the same existing protein. Unfortunately, with no relevant BLAST hit in whichever kingdom of life (probably due to lack of relevant sequence databases), this mysterious finding did not allow any clues as to the role of protein glycosylation in a unicellular organism.

### DNA-Based Characterization and Phylogeny

To complement the structural data on *N*-glycans, phylogenetic relations were examined between the two glyco groups with the help of ITS1-5.8S-ITS2 rDNA barcodes. Genomic DNA was extracted from live samples, as well as products, and a green alga-specific primer pair was used to amplify the aforementioned region. Just like the live strain SAG211-34 (GenBank MN194596), the ‘‘Raa’’ samples yielded a single sequence that could be Sanger sequenced directly. The homology between ‘‘Raa’’ samples and SAG211-34 was 100% throughout the whole DNA fragment. This was exactly the same sequence as the GenBank entry MK248017, which describes FACHB-31. A BLAST search with this sequence retrieves a closest entry assigned to an *Auxenochlorella pyrenoidosa* isolate (KM514847). Notably, SAG211-34 is sometimes designated as *Chlorella sorokiniana*^1^ (Wu et al., 2018), a species name already used for the type strain SAG211-8k that displays clearly different *N*-glycans (Mocsai et al., 2020b), whereas FACHB-31 is described as *Chlorella* species^2^. Elsewhere, FACHB-31 was termed *S. obliquus* (Xu et al., 2015). This clearly calls for the attention of taxonomist for a well-needed reclassification event.

**FIGURE 8:**
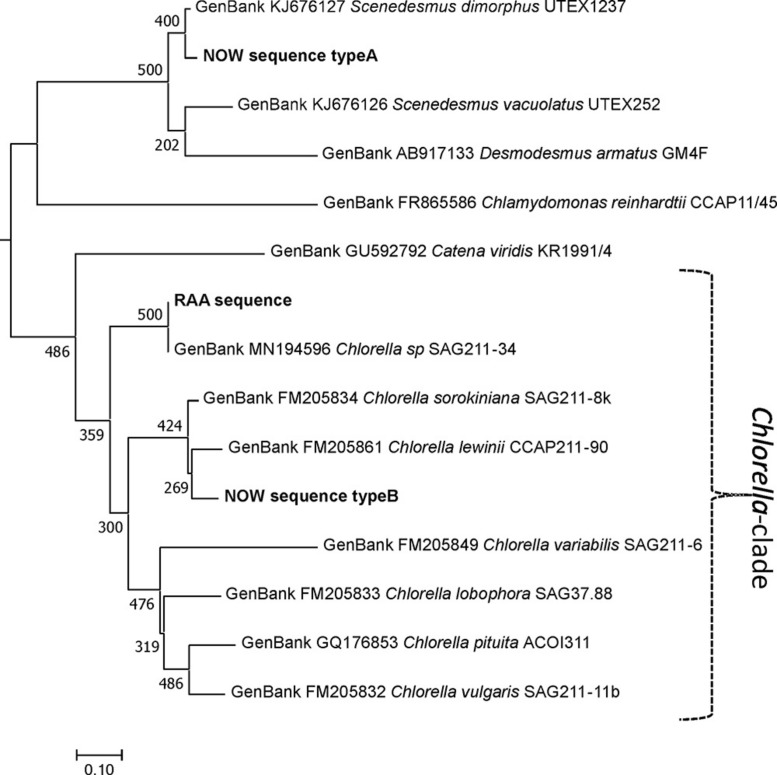
Phylogeny of the ITS1-5.8S-ITS2 rDNA fragment of different microalga species. Experimentally obtained sequences (marked bold) were aligned with database entries; GenBank accession numbers are provided for each of these. Sequences were aligned using the MAFFT alignment tool with the algorithm L-INS-i (Katoh and Standley, 2013), and phylogeny was inferred using PhyML (Guindon et al., 2010). Branch support was calculated by 500 bootstrap repetitions. The tree was rooted on midpoint and visualized in MEGA7 (Kumar et al., 2016). Scale bar: base substitutions per site.

Sample “Now” required subcloning into *Escherichia coli* cells. The results proved that the sample was indeed a mixture. Of 17 clones chosen, 16 almost identical clones were related to species of Scenedesmaceae [e.g., *Scenedesmus dimorphus* (UTEX417 and UTEX1237) or *Acutodesmus obliquus* (SAG276-10)]. One clone was related to *Chlorella lewinii* (strain CCAP211-90). Given the large number of Scenedesmaceae-related clones, these almost certainly account for the dominant features of the “Now” glycan pattern. *Scenedesmus* and *Chlorella* belong to different classes of green algae. Although the two glyco groups are both characterized by a dominating 1,049.3 peak [M+Na]^+^, their genetic distance is profound as visualized in Figure 8. This genetic unrelatedness explains why the isobaric *N*-glycans have such different structures.

## Discussion

The analysis of *N*-glycans from arbitrarily selected *Chlorella* live strains and *Chlorella* products has revealed an astonishing diversity of glycan structures (Mocsai et al., 2020a). In many cases, the masses of the dominant peaks deviate and allow an immediate discrimination of strains on the basis of a simple MALDI spectrum. In some cases, the “Sol” and “Jar” (and related) groups occur, even though the underlying structure is possibly different (Mocsai et al., 2020a). The same holds true for the “Raa” and “Now” samples with their common major glycan at *m/z* = 1,049.3 [M+Na]^+^. Subtle differences of the glycan patterns aroused our suspicion and revealed that these samples are derived from rather different microalgal sources. While DNA barcoding places the “Raa” strain clearly in the *Chlorella* clade, the “Now” glycan pattern appears to belong to the family of Scenedesmaceae, which is phylogenetically very remote to *Chlorella* but rather related to *Chlamydomonas*. This common root may be the cause for both the “Now” C-5 sample and *Chlamydomonas reinhardtii* carrying xylose in β1,2-linkage to the core’s β-mannosyl residue (Mathieu-Rivet et al., 2013; Lucas et al., 2020). Apart from this residue being 3-*O*-methylated in “Now”, this xylose residue is also found in all land plants (Bardor et al., 1999; Altmann, 2007). The position of the second xylose in C-5 “Now”, however, does not remind of the other two types of xylosyl residues proposed as being β1,4-linked to α-mannosyl residues (Lucas et al., 2020). A 3-*O*-methyl xylose was, however, found in a proteoglycan of the red alga *Rhodella grisea* (Capek et al., 2008). The major species of both microalgal glyco types contained just one methyl group and two pentoses as additions to the common *N*-glycan core but nevertheless exhibited an astounding degree of difference. This example underpins the necessity of a rather close look at the glycan patterns if they are to be used for species differentiation.

Hoping that knowledge of the proteins carrying these novel complex-type *N*-glycans would give hints about their functional role, we aimed at identifying proteins carrying these *N*-glycans. This effort led to the identification of two glycopeptides in the eponymous “Raa” sample C-55. Much to our surprise, both glycopeptides and another peptide aligned perfectly with a hypothetical protein postulated in the course of a rhizobial genome project. The respective gene segment in turn retrieved an identical sequence in an *A. pyrenoidosa* genome. No clue as to the function of this protein arose from this finding as no homologous protein could be found in plants or bacteria (including *archaea*)—not even in the otherwise highly homologous genome of *Auxenochlorella protothecoides*. It may be added here that growing of SAG211-34 in the presence or absence of antibiotics (ampicillin and tetracycline) did not alter the *N*-glycan pattern.

Our ongoing analysis of microalgal *N*-glycans has by now revealed 13 different glycan patterns as published recently (Mocsai et al., 2020a). After adding live *Chlorella* strains and not yet published unique patterns, the number is already above 20. This diversity arises from an even greater number of glyco-enzymes. So far, every analysis of a particular structure unveiled ever new features and thus glyco-enzymes unique for the particular strain. It will be exciting to find out how inventive and diverse microalgae actually can be. At any rate, the results surfaced by the recent experiments should raise the attention of taxonomists. An *N*-glycan-based characterization of microalgae appears to open a chance for identical strains to get together under one taxonomic roof despite traditionally different names. Vice versa, differing strains that have falsely been given the same name simply because microalgae are notoriously poor in distinctive and reliably presented optical features can be told apart with certainty.

## Data Availability Statement

The datasets presented in this study can be found in online repositories. The names of the repository/repositories and accession number(s) can be found in the article/Supplementary Material.

## Author Contributions

RM carried out most of the wet-chemical work including molecular biology and phylogeny. HK, MB and PK conducted and analyzed the NMR experiments. JS performed the glycoproteomic work. FA conceived and supervised the study, analyzed the data, and wrote the manuscript. All authors contributed substantially to the work and approved the submitted version.

## Conflict of Interest

The authors declare that the research was conducted in the absence of any commercial or financial relationships that could be construed as a potential conflict of interest.

## Footnotes

1 sagdb.uni-goettingen.de/

2 algae.ihb.ac.cn/english
