# Supplementary material for: The Structural Difference of Isobaric N-Glycans of Two Microalgae Samples Reveals Taxonomic Distance
Source: Front Plant Sci. 2021 Apr 26;12:643249. doi: 10.3389/fpls.2021.643249 (PMC8107433; doi:10.3389/fpls.2021.643249)
Supplement: Supplementary file 1 [file Data_Sheet_1.PDF]

Supplementary Material

to

The structural difference of isobaric N-glycans of two microalgae samples reveals taxonomic distance

Réka Mócsai[1]**, Hanspeter Kaehlig**[2]**, Markus Blaukopf**[1]**, Johannes Stadlmann**[1]**, Paul Kosma**[1]**, Friedrich Altmann**[1]

[1] Department of Chemistry, University of Natural Resources and Life Sciences Vienna, Vienna, Austria

[2] Department of Organic Chemistry, Faculty of Chemistry, University of Vienna, Austria *****

Correspondence: Friedrich Altmann; friedrich.altmann@boku.ac.at

Supplementary Tables

Table S1 * List of products considered in this study. We have no information about the producers and several products may stem from the same source. However, physical appearance and dissolution behavior argue for certainly more than one source. CV: *Chlorella vulgaris*; CP: *Chlorella pyrenoidosa*

Internal #	Label	Species	Contact	Vendor´s domicile	Date of purchase
**Glyco-type 'Raa'**					
6	Green foods	-	greenfoods.com	USA / Ontario CA	3/2018
55	Raab	-	raabvitalfood.com	GER / Rohrbach	9/2018
60	Ehn	CV	eder-health-nutrition.de	GER / Breisach	9/2018
63	Bio Prophyl	CP	bioprophyl.de	GER / Nitz	9/2018
64	Tierra Verde	-	tierra-verde.de	GER / Reutlingen	9/2018
68	Medicura	CP	medicura.com	GER / Burglauer	9/2018
70	Greenfood	-	greenfood-shop.de	NED / Groningen	9/2018
76	Amlawell	-	amlawell.de	GER / Kiel	9/2018
95	Mosaik	CV	my-mosaik.de	D-Wertheim	1/2020
96	Flügelschwinger	-	fluegelschwinger.com	D-Hamburg	1/2020
98	Mr. Brown	CV	mister-brown.com	D-Schillingsfürst	1/2020
102	Natura forte	CV	NaturaForte.de	D-Brüggen	1/2020
110	Now (new batch)	-	nowfoods.com	USA/ Bloomingdale IL	10/2019
**Glyco-type 'Now'**					
5	Now	-	nowfoods.com	USA/ Bloomingdale IL	3/2018

Table S2 ∗ ^{1}H and ^{13}C chemical shifts (δ, ppm) and in parentheses J couplings (Hz) for the glycan preparation 'Raa'

Sugar Residue	β-Ara*f*-(1→	→4)-α-(3-OMe)-Man*p*-(1→	→3)-β-Man*p*-(1→	→3,4)-β-Glc*p*NAc-(1→	α-Araf-(1→	→4)-GlcolNAc
H1	5.300 (4.8)	5.120 (2.0)	4.819 (0.6)	4.666 (7.8, br) [1)]	5.219 (1.5)	a 3.630 (11.7, 6.6) [2)]
						b 3.717 (11.7, 4.9)
C1	104.51 (179.4)	105.00 (172.5)	101.62 (161.2)	103.42 (166.1)	110.69 (176.1)	63.77
H2	4.101 (4.8, 8.2)	4.293 (2.0, 3.1)	4.201 (0.6, 3.3)	3.880 (7.8, 9.3)	4.054 (1.6, 3.4)	4.211 (6.6, 4.9, 6.3)
C2	78.74	68.57	72.86	58.31	83.94	55.55
H3	3.980 (8.2, 7.6)	3.743 (3.1, 9.4)	3.752 (3.3, 9.4)	3.894 (9.3, 8.8)	3.945 (3.4, 5.9)	3.911 (6.3, 3.4)
C3	76.69	82.77	83.64	79.56	79.15	70.72
H4	3.823 (7.6, 6.9, 3.1)	3.769 (9.4, 9.7)	3.690 (9.4, 9.8)	3.905 (8.8, 9.8)	4.342 (5.9, 4.9, 3.6)	3.808 (3.4, 6.3)
C4	83.88	75.50	68.46	76.95	86.63	81.83
H5	a 3.664 (6.9, 12.0)	3.891 (9.7, 6.4, 2.2)	3.390 (9.8, 5.9, 2.3)	3.529 (9.8, 3.9, 2.2)	a 3.682 (4.9, 12.1)	3.855 (6.3, 6.6, 3.3)
	b 3.801 (3.1, 12.0)				b 3.775 (3.6, 12.1)	
C5	65.35	74.50	79.04	77.55	63.54	73.96
H6		a 3.775 (6.4, 12.0)	a 3.748 (5.9, 12.1)	a 3.742 (3.9, 12.4)		a 3.543 (6.6, 11.7)
		b 3.948 (2.2, 12.0)	b 3.910 (2.3, 12.1)	b 3.922 (2.2, 12.4)		b 3.759 (3.3, 11.7)
C6		63.80	63.40	62.76		64.56
C<u>H</u>$_3$		3.424		2.064		2.046
<u>C</u>H$_3$		58.76		24.94		24.86
CO				177.27		177.09

[1)] br broad signal [2)] a,b geminal protons

Table S3 ∗ ^{1}H and ^{13}C chemical shifts (δ, ppm) and in parentheses J couplings (Hz) for the glycan preparation 'Now'

Sugar Residue	α-Man*p*-(1→	→2,6)-β-Man*p*-(1→	β-(3-OMe)-Xylp-(1→	→3,4)-β-Glc*p* NAc-(1→	β-Xylp-(1→	→4)-Glc*p*NAc
H1	4.908 (1.8)	5.006 (1.0)	α 4.559 (7.8) [1)]	α 4.599 (7.5, br) [2)]	α 4.523 (7.7)	α 5.179 (2.5)
			β 4.557 (7.8)	β 4.588 (7.5, br)	β 4.522 (7.7)	β 4.687 (8.2)
C1	102.33 (172.8)	100.03 (159.2)	106.65 (166.0)	103.43	α 105.51 (163.0)	α 93.16 (172.0)
					β 105.49 (163.0)	β 97.52 (161.5)
H2	3.998 (1.8, 3.5)	4.221 (1.0, 3.2)	3.433 (7.8, 9.4)	3.929 (7.5, 9.8, br)	α 3.247 (7.7, 9.4)	α 3.875 (2.5, 9.8)
					β 3.245 (7.7, 9.4)	β 3.697 (8.2, 9.8)
C2	72.61	80.75	75.36	58.43	75.63	α 56.37 / β 58.84
H3	3.812 (3.5, 9.1)	3.595 (3.2, 9.7)	3.227 (9.4, 9.2)	α 4.297 (9.8, 8.8, br)	α 3.417 (9.4, 9.2)	α 3.877 (9.8, 8.8)
				β 4.284 (9.8, 8.8, br)	β 3.416 (9.4, 9.2)	β 3.675 (9.8, 8.8)
C3	73.31	74.92	87.61	77.52	78.29	α 71.96 / β 75.21
H4	3.656 (9.1, 9.7)	3.622 (9.7, 9.6)	3.716 (9.2, 10.1, 5.7)	α 3.918 (8.8, 9.8)	3.613 (9.2, 10.1, 5.7)	α 3.632 (8.8, 9.5)
				β 3.913 (8.8, 9.8)		β 3.624 (8.8, 9.5)
C4	69.38	69.51	71.46	78.12	72.08	α 82.21 / β 81.77
H5	3.635 (9.7, 5.2, 2.2)	3.532 (9.6, 2.1, 4.6)	a 3.255 (10.1, 11.6) [3)]	α 3.818 (9.8, 4.7, 2.4)	a 3.260 (10.1, 11.6)	α 3.870 (9.5, 4.5, 2.2)
			b 3.942 (5.7, 11.6)	β 3.809 (9.8, 4.7, 2.4)	b 3.938 (5.7, 11.6)	β 3.508 (9.5, 5.8, 2.2)
C5	75.46	77.18	67.69	77.07	67.80	α 72.68 / β 77.25
H6	a 3.752 (5.2, 12.2)	a 3.741 (2.1, 11.3)		a α 3.843 (4.7, 12.2)		a α 3.670 (4.5, 12.1)
	b 3.875 (2.2, 12.2)	b 3.948 (4.6, 11.3)		a β 3.836 (4.7, 12.2)		a β 3.647 (5.8, 12.4)

				b α 3.963 (2.4, 12.2)	b α 3.780 (2.2, 12.1)
				b β 3.960 (2.4, 12.2)	b β 3.824 (2.2, 12.4)
C6	63.60	68.23		63.34	α 62.72 / β 62.81
$C\underline{H}_3$			3.612	α 2.061 / β 2.059	α 2.029 / β 2.027
$\underline{C}H_3$			62.55	24.96	α 24.63 / β 24.85
CO				177.17	α 177.27 / β 177.43

[1] α,β reducing sugar α or β [2] br broad signal [3] a,b geminal protons

Table S4 ⁎ Masses of glycopeptides and peptides in the ESI-MS analysis of 'Raa' C-55

Peptide	Glycoform	Exp. Mass of 2+	Calc. mass of 2+
VNVVNNTLISVNQK	os2210	1202.588	1202.5857
VNVVNNTLISVNQK	os2211	1209.596	1209.5935
VNVVNNTLISVNQK	os2221	1275.667	1275.6146
VNVVNNTLISVNQK	deglycosylated	771.928	771.9362
FANLTSTVDELAK	os2210	1136.024	1136.0271
FANLTSTVDELAK	os2211	1143.029	1143.0271
FANLTSTVDELAK	os2221	1209.053	1209.0482
FANLTSTVDELAK	deglycosylated	705.380	705.3698
VPELIDDLAALQAAVQPLLNNR	No glycopeptide		1187.1568

Supplementary Figures

Figure S1 ∗ Higher molecular weight range of the MALDI spectra of a Raa and a Now sample showing less abundant pentose-containing glycans (complementary to **Figure 1**). While both glycomes contain structures with one and two pentoses plus methyl groups, the fine structure differs. It should be added that for none of the two samples can be guaranteed that these minor glycans arise from the same algal strain as the major glycan structures even though the continuity of the hexose - pentose - methyl series (notice the absence vs. presence of 2220 and 3220 peaks in Raa and Now, respectively) may suggest so. *Subtle quantitative differences compared to Fig. 1 result from different preparations or samples (e.g. Raa).*

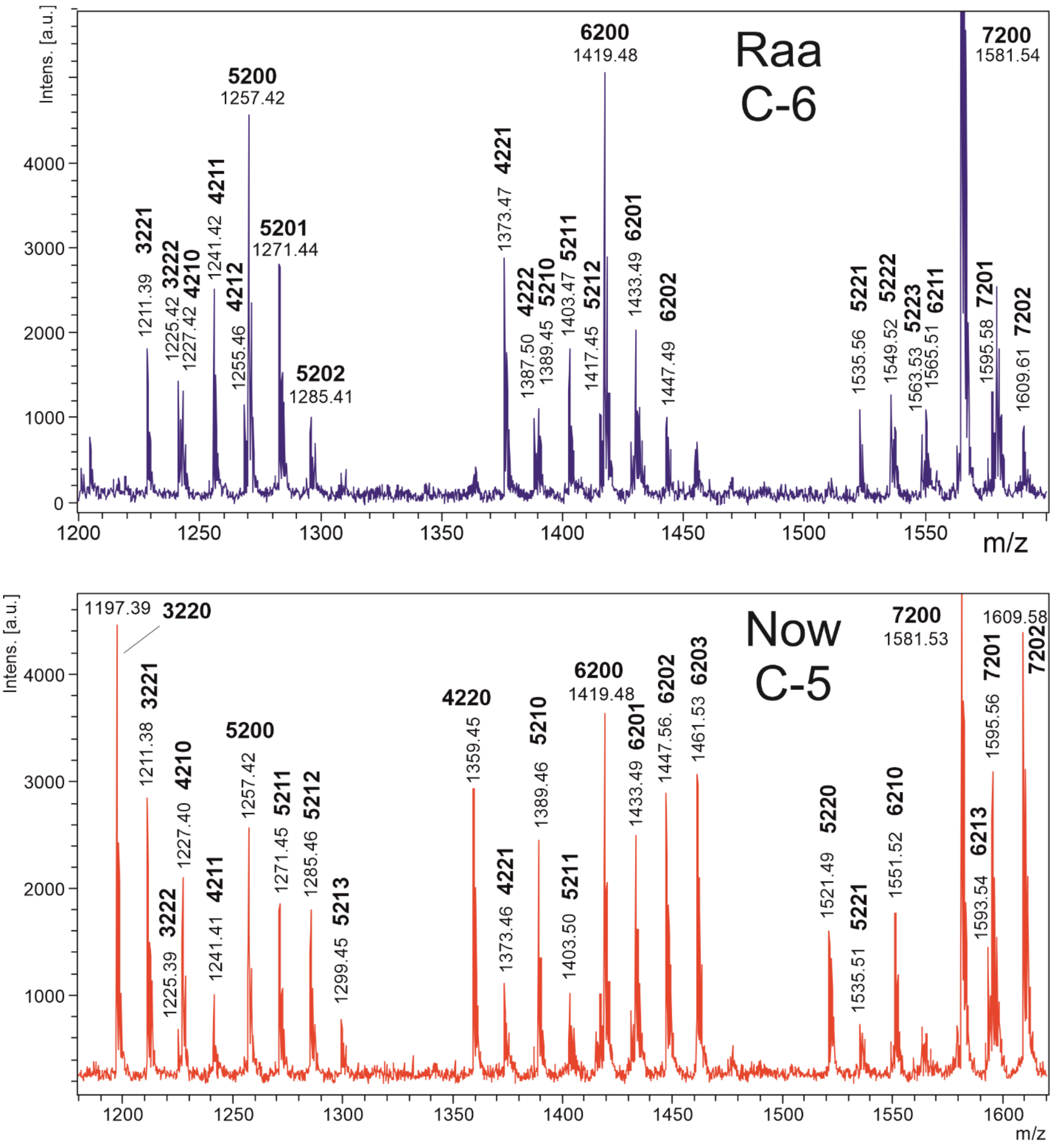

Figure S2 ∗ Linkage analysis by permethylation and GC-MS of partially methylated alditol acetates. Upper panel: GC-MS traces. Lower panels: Spectra of relevant peaks showing methyl-, deuteromethyl- and natural methyl groups in different colors.

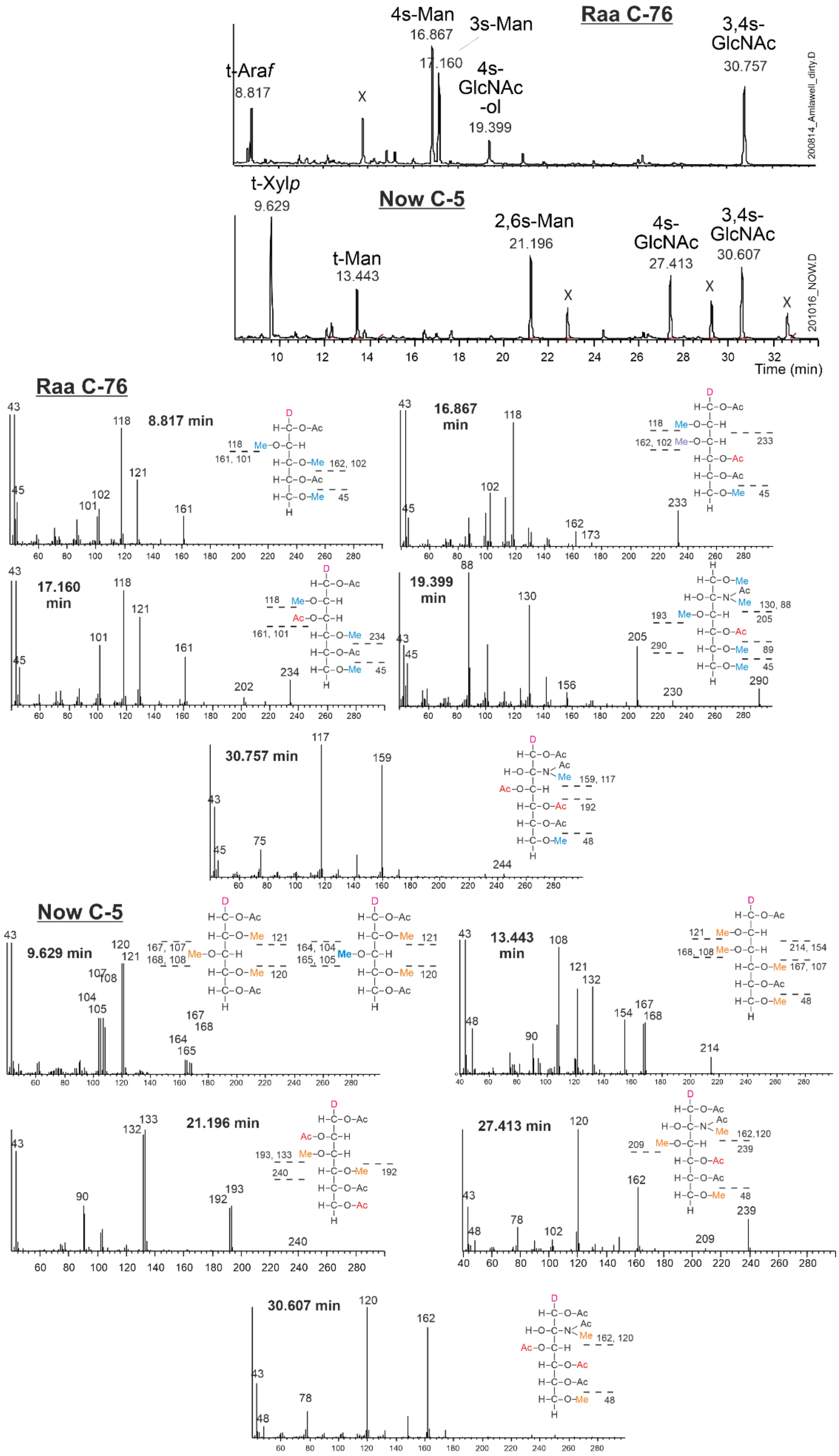

Figure S3 ∗ Proof of terminal α-mannosidic residues in 'Now' N-glycans. The complex-type N-glycans of Now C-5 formed a series of glycans with up to five hexose residues. Treatment (+) of selected HILIC fractions with α-mannosidase shows that all terminal hexoses from os5220 are α-mannosyl residues (A- and A+) and that os3220 can be degraded down to the β-mannosyl- residue (B- and B+). Thus, the five mannose residues were possibly arranged as shown in the cartoon. Besides, the panels show that methylation hinders degradation of oligomannosidic N-glycans.

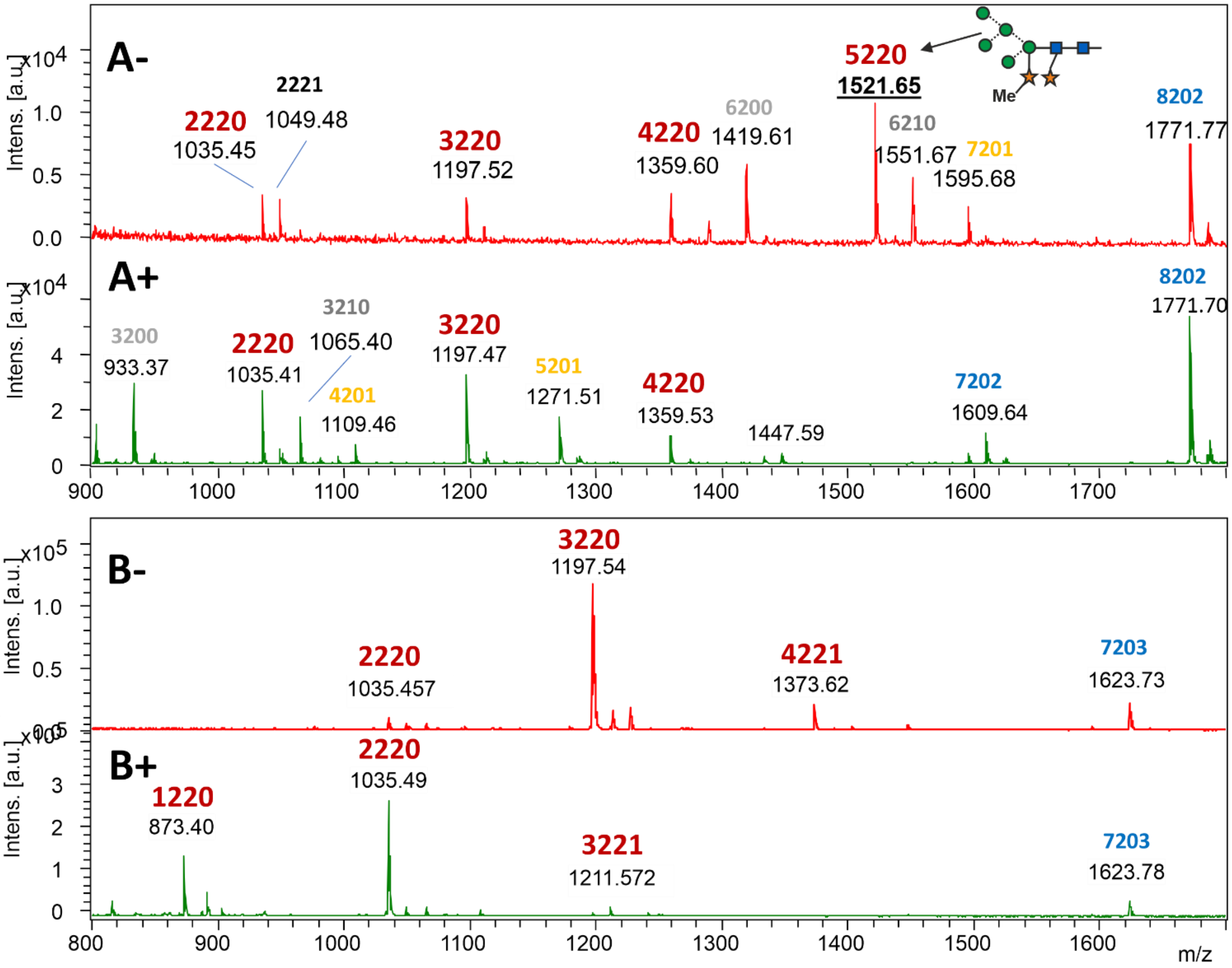

Figure S4 ∗ HSQC spectrum of glycan preparation 'Raa' C-76 (excluding CH_3 of acetyl groups). Top trace ^{1}H NMR spectrum, left trace ^{13}C DEPTq 135 spectrum.

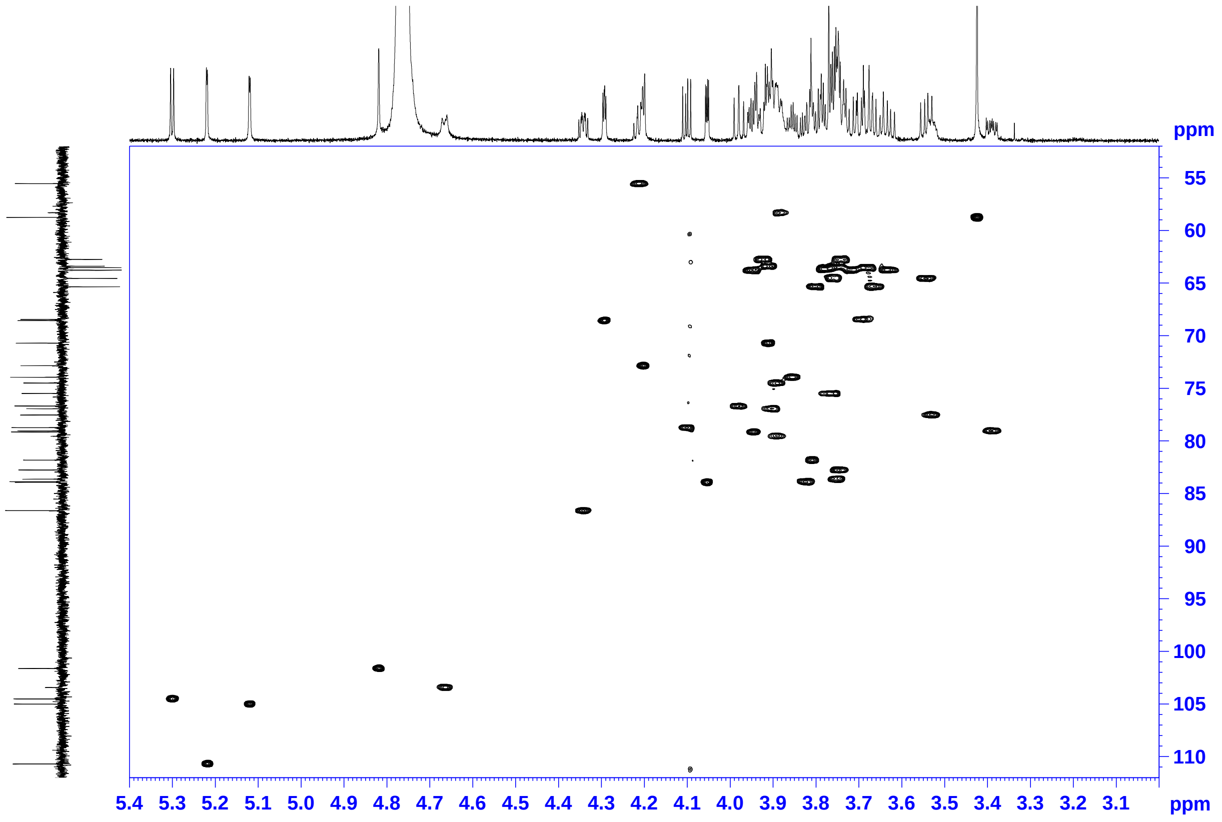

Figure S5 ∗ HSQC spectrum of glycan preparation 'Now' C-5 (excluding CH_3 of acetyl groups). Top trace ^{1}H NMR spectrum, left trace ^{13}C DEPTq 135 spectrum.

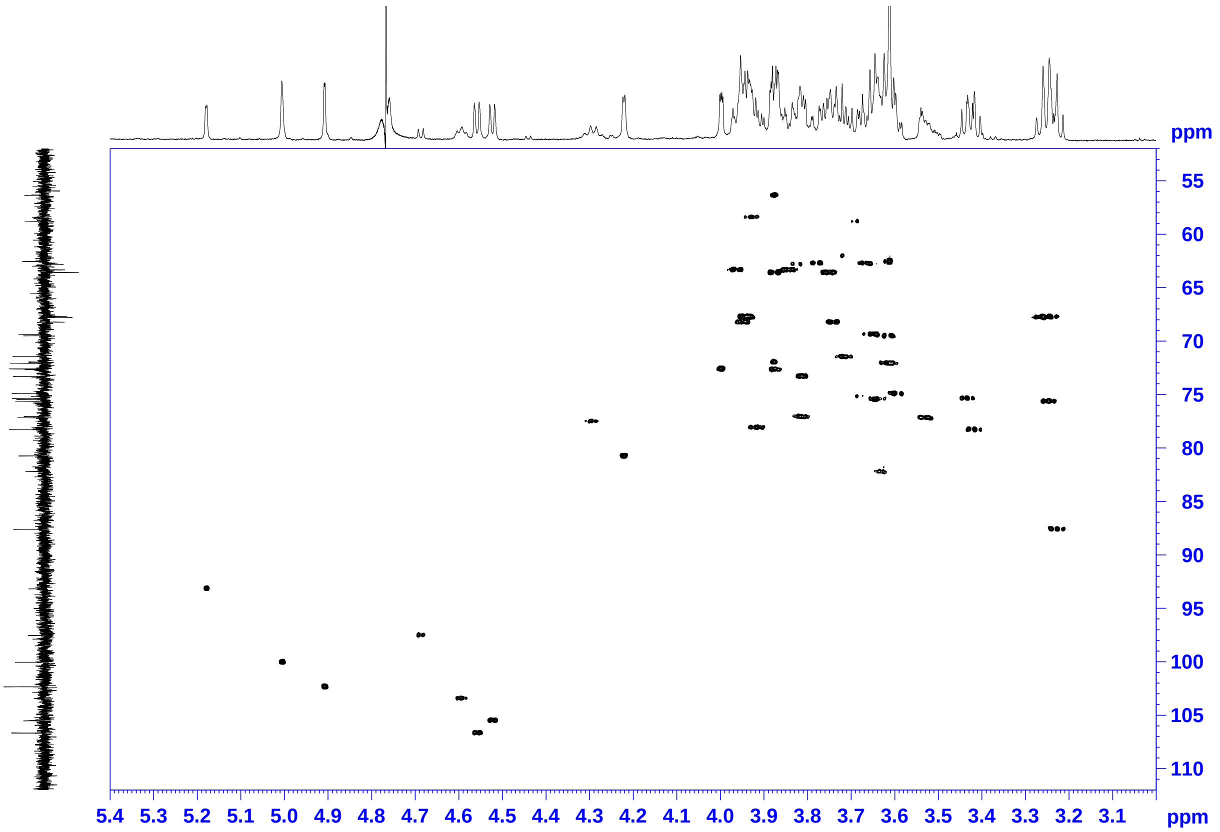

Figure S6 ∗ Three selected sugar spin systems of glycan preparation ‘Raa’ C-76. Bottom trace: experimental 1D TOCSY spectrum; top trace: calculated spectrum derived from a spin simulation.

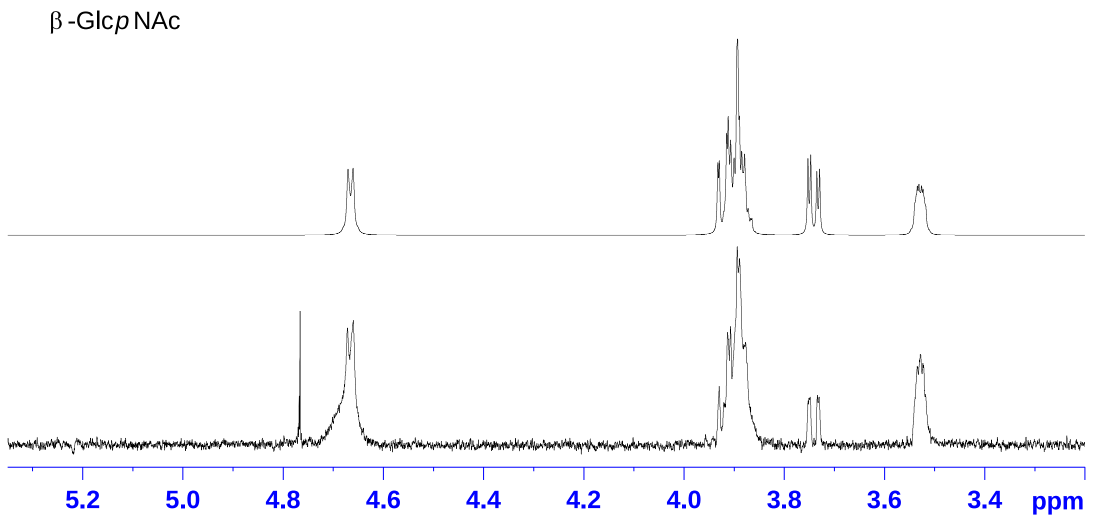

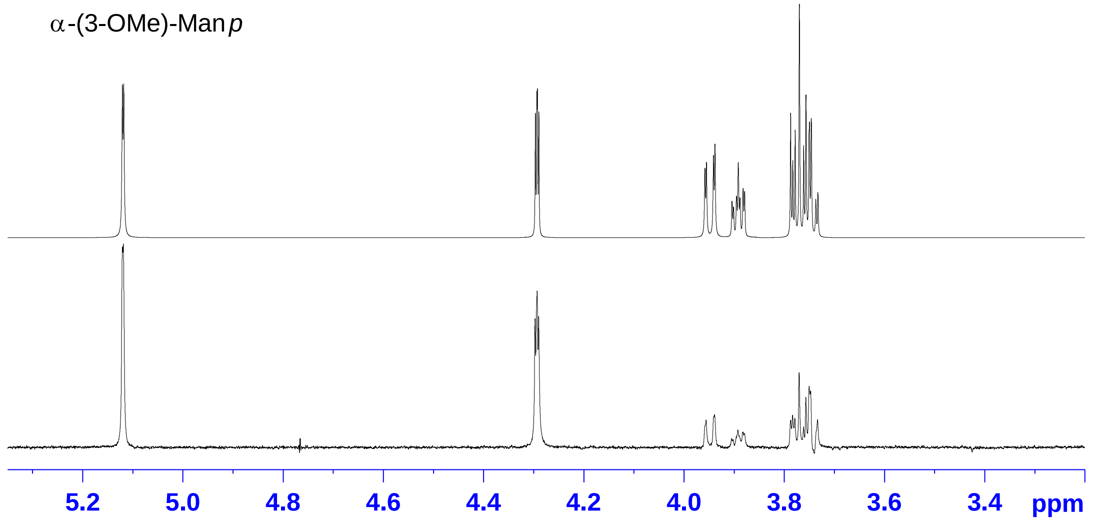

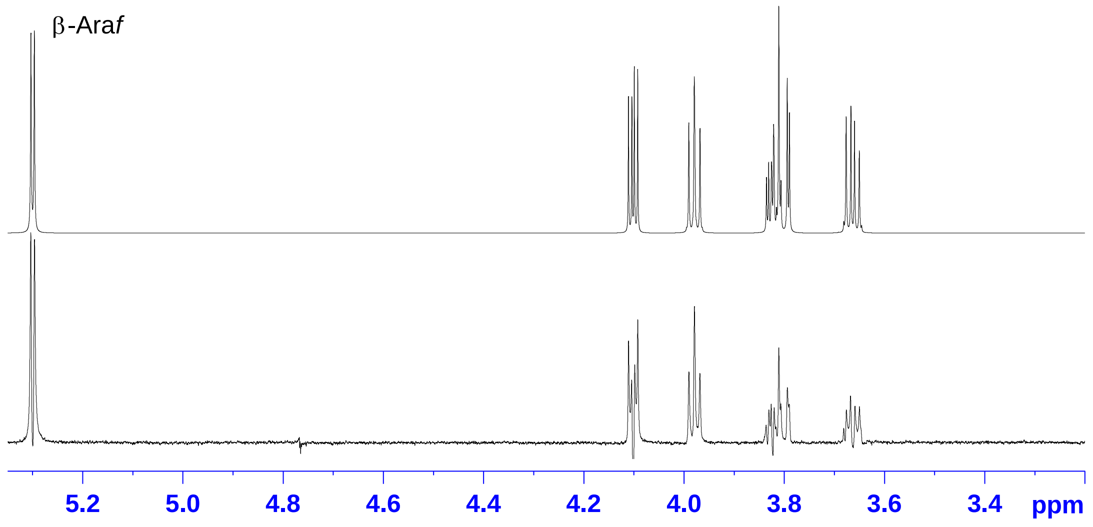

Figure S7 ∗ Three selected sugar spin systems of glycan preparation 'Now' C-5. Bottom trace: experimental 1D TOCSY spectrum; top trace: calculated spectrum derived from a spin simulation.

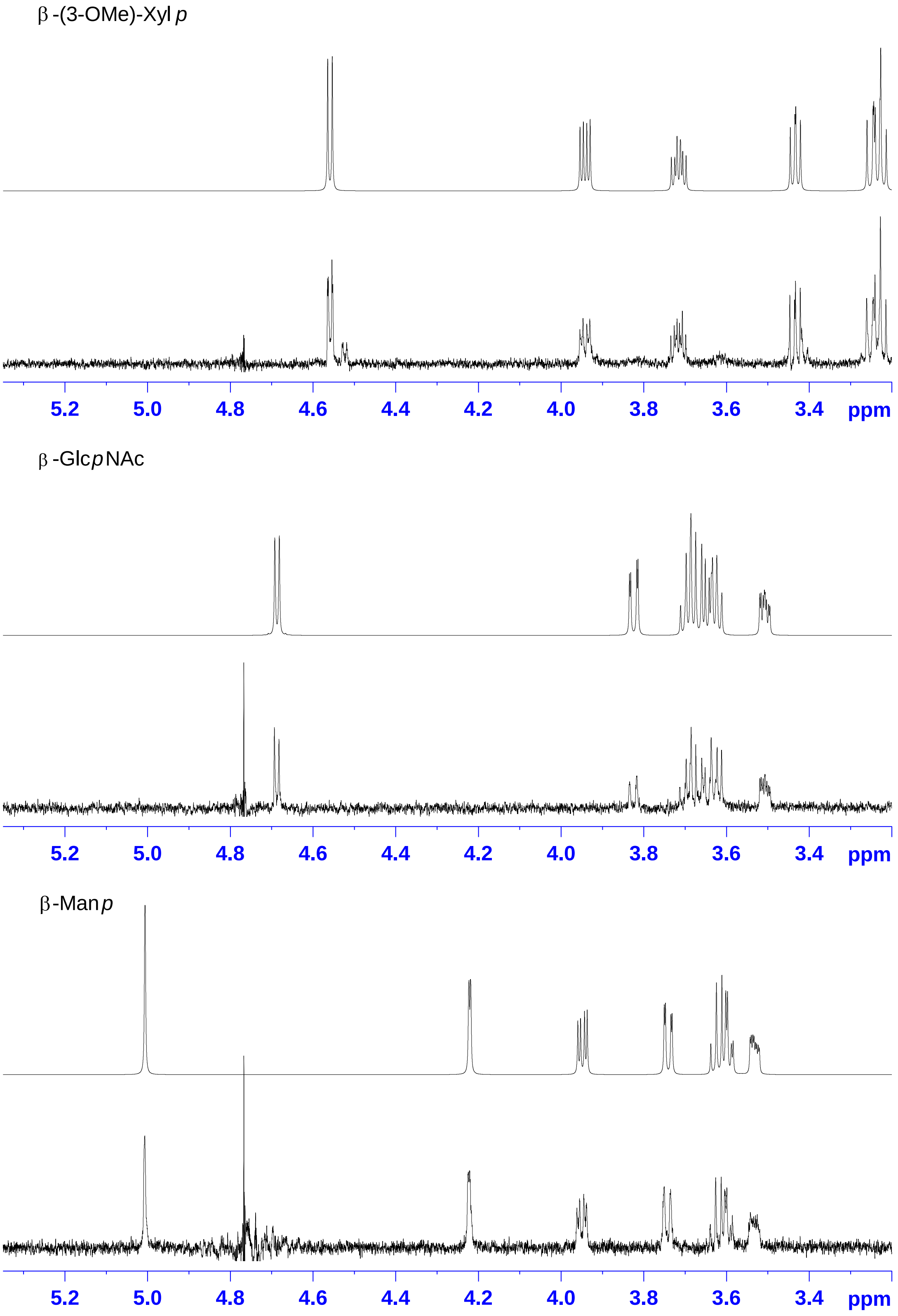

Figure S8 ∗ Structure of glycan preparation ‘Raa’ C-76

α-(3-OMe)-Man*p*-(1→3)-β-Man*p*-(1→4)-β-GlcpNAc-(1→4)-GlcolNAc

β-Ara*f*-(1→4)┘ (on α-(3-OMe)-Man*p*)   α-Ara*f*-(1→3)┘ (on β-GlcpNAc)

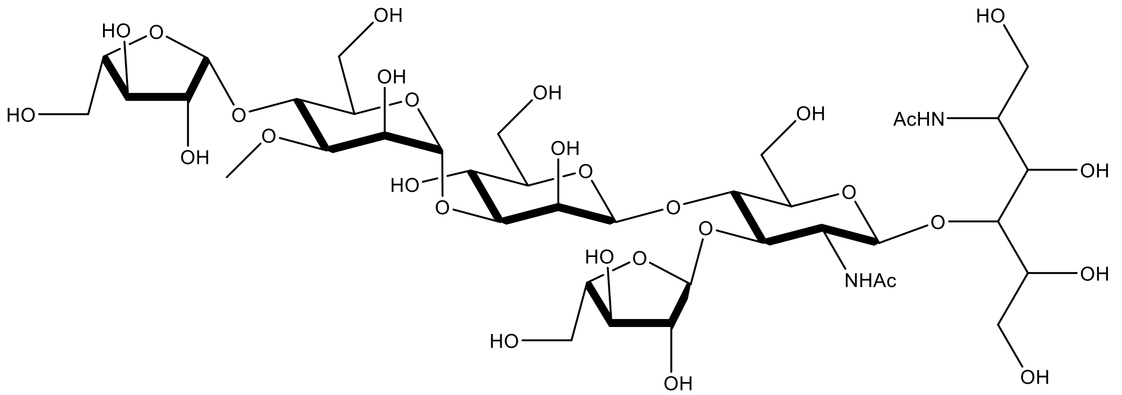

CFG cartoon

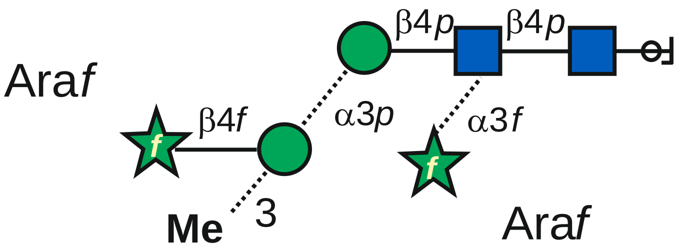

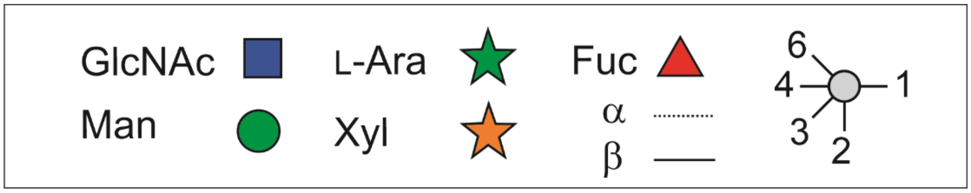

Figure S9 ∗ Structure of glycan preparation 'Now' C-5

α-Man*p*-(1→6)-β-Man*p*-(1→4)-β-GlcpNAc-(1→4)-α/β-Glc*p*NAc

β-(3-OMe)-Xyl*p*-(1→2)┘ β-Xyl*p*-(1→3)┘

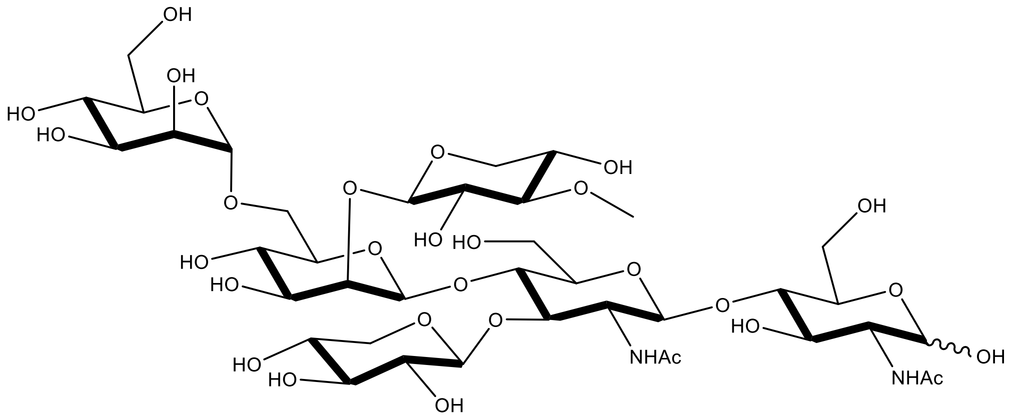

CFG cartoon

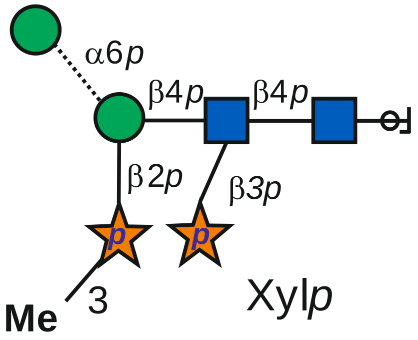

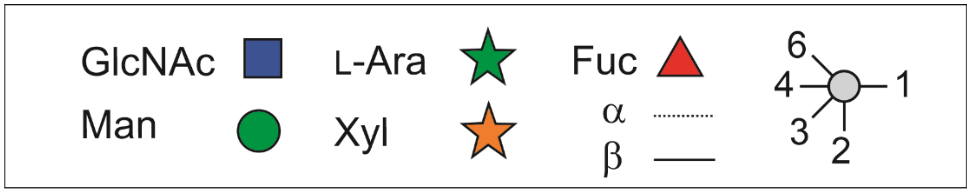

Figure S10 ∗ MSMS spectrum of a peptide from the glycoprotein found in Raa C-55 (Uniprot A0A0N1KWP3)

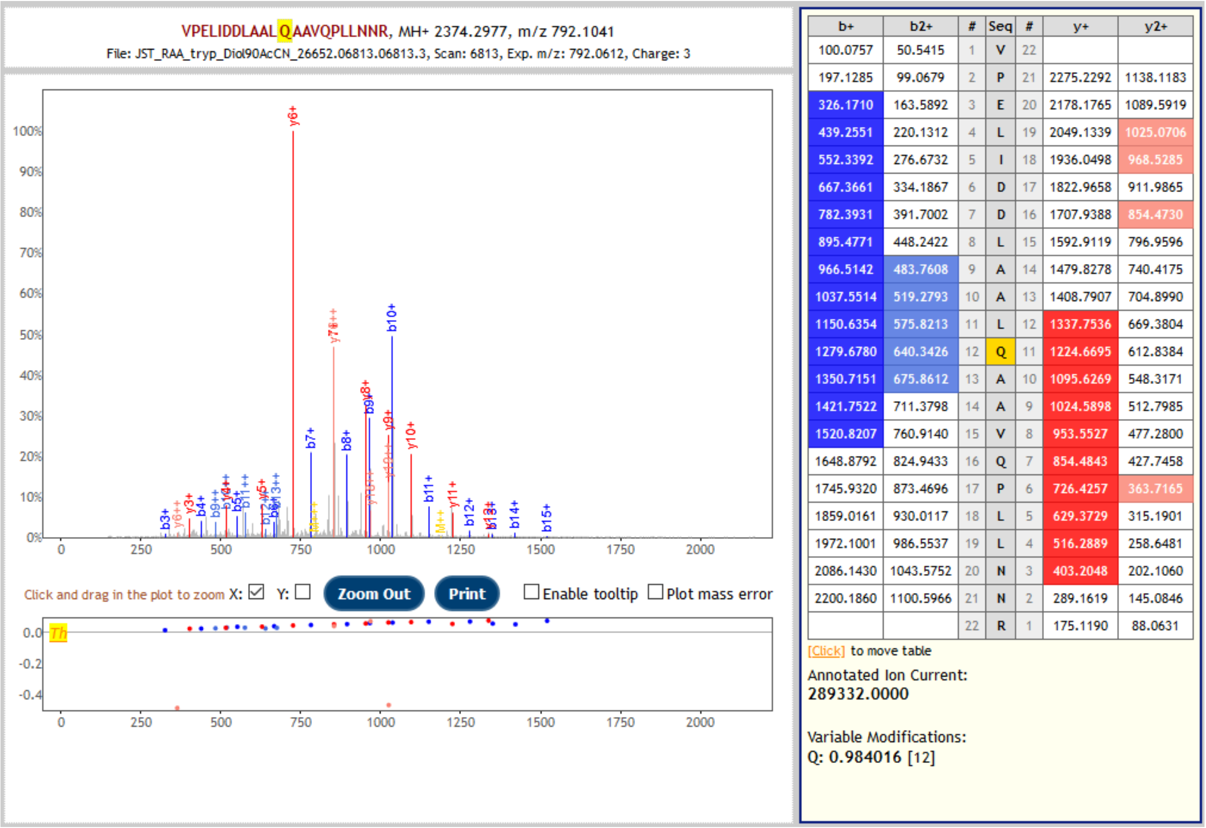

b+	b2+	#	Seq	#	y+	y2+
100.0757	50.5415	1	V	22		
197.1285	99.0679	2	P	21	2275.2292	1138.1183
326.1710	163.5892	3	E	20	2178.1765	1089.5919
439.2551	220.1312	4	L	19	2049.1339	1025.0706
552.3392	276.6732	5	I	18	1936.0498	968.5285
667.3661	334.1867	6	D	17	1822.9658	911.9865
782.3931	391.7002	7	D	16	1707.9388	854.4730
895.4771	448.2422	8	L	15	1592.9119	796.9596
966.5142	483.7608	9	A	14	1479.8278	740.4175
1037.5514	519.2793	10	A	13	1408.7907	704.8990
1150.6354	575.8213	11	L	12	1337.7536	669.3804
1279.6780	640.3426	12	Q	11	1224.6695	612.8384
1350.7151	675.8612	13	A	10	1095.6269	548.3171
1421.7522	711.3798	14	A	9	1024.5898	512.7985
1520.8207	760.9140	15	V	8	953.5527	477.2800
1648.8792	824.9433	16	Q	7	854.4843	427.7458
1745.9320	873.4696	17	P	6	726.4257	363.7165
1859.0161	930.0117	18	L	5	629.3729	315.1901
1972.1001	986.5537	19	L	4	516.2889	258.6481
2086.1430	1043.5752	20	N	3	403.2048	202.1060
2200.1860	1100.5966	21	N	2	289.1619	145.0846
		22	R	1	175.1190	88.0631

Supplementary Data

Nucleotide sequence of the ITS1-5.8S-ITS2 barcode region of the ‘Raa’ glycotype sample C-76 and the 100% homologous live strain SAG211-34. This sequence has been deposited in GenBank (MN194596) as *Chlorella sp.* and is identical to MK248017 assigned to strain FACHB-31.

>RAA_sequence

ACACACCGCCCGTCGCTCCTACCGATTGGGTGTGCTGGTGAAGTGTTCGGATTGGCGACCGGGTGCGGTCT
CCGCTCTCGGCCGCCGAGAAGTTCATTAAACCCTCCCACCTAGAGGAAGGAGAAGTCGTAACAAGGTTTCC
GTAGGTGAACCTGCGGAAGGATCATTGAATCGATCGAATCCACTCTGTGAACCAAACGTCCCCCCTTGGGT
GCGGGCTTCGGTCTGCCCCAAGGCGTCGGTTCCCTGGCTGGGGTCTTCGGACCGCAGTTAGGTCCGGCGGG
CGCGCCCTCTGGCGTGTCGGCCCTCGTGGCTGCCGCCAGTTGGGTTCGCTGGAAATTGTATCCAACTCAACC
CACCCCAAACCACAACTTATACTGAAGCAATCGGTGAGTGCACTCTGGTGCCTCGCTCTAACCAAAGACAA
CTCTCAACAACGGATATCTTGGCTCCCGTATCGATGAAGAACGCAGCGAAATGCGATACGTAGTGTGAATT
GCAGAATTCCGTGAACCATCGAATCTTTGAACGCAAATTGCGCCCAAGGCTTCGGCCGAGGGCATGTCTGC
CTCAGCGTCGGCTTACCCCCTCGCTCCCCCTCTCCTTTGGAGTGGGTGAACGGATCTGGTTTTCCCGGCTAC
GTGCTTCTGCACGCCCGGGTTGACTGAAGTGTAGAGGCTTGAGCATGGACCCCGTTTGTAGGGCAATGGCT

TGGTAGGTAGCTTAGCTACACCGCCTGCCGTGGTCCGAGGGGACTTTGCTGGCGGCCCAGCAGGAATTCGG
GTGTTGGGTTTCCCACCCCGAAAGCTTCAAACCTTCGACCTGAGCTCAGGCAAGA

Nucleotide sequences of the ITS1-5.8S-ITS2 barcode region of the clones acquired from Now C-5 sample. Type A clones (94%) are closely related to *Scenedesmus dimorphus* (strain UTEX1237) while the single Type B clone (6%) was related to *Chlorella lewinii* (strain CCAP211-90).

>NOW_sequence_typeA_clone8

ACACACCGCCCGTCGCTCCTACCGATTGGGTGTGCTGGTGAAGTGTTCGGATTGGCAGCTTAGGGTGGCAA
CACCTCAGGTCTGCCGAGAAGTTCATTAAACCCTCCCACCTAGAGGAAGGAGAAGTCGTAACAAGGTTTCC
GTAGGTGAACCTGCGGAAGAATCATTGAATTATTAAAACCACAATGTGAACCTTATTGTTCCGTGCCTTTGC
TGCCGGCAAGGCAATCAGCTTTGCCTGATTGTACTTGCAAGCTGGTGCGAGTTTTATACTTGCATCAGTGGC
GCTCTGGCATGCTTATACACCAGTGCTAACCACTGTCAAAACCAAACTCTGAAGCTTTGATTGCTATTAATT
GGCAATCTTAACCAAAGACAACTCTCAACAACGGATATCTTGGCTCTCGCAACGATGAAGAACGCAGCGA
AATGCGATACGTAGTGTGAATTGCAGAATTCCGTGAACCATCGAATCTTTGAACGCATATTGCGCTCGAGC
CCTCGGGCAAGAGCATGTCTGCCTCAGCGTCGGTTTATAACCTCACCCCTCTCTCCTTTTGGAGAGCTGGTT
AGCTTCTAGCTGGCCTTAGGAGTGGATCTGGCTTTCCCATTTGGTTTATTCTGAATGGGTTGGCTGAAGCTT
AGAGGCTTAAGCAAGGACCCGATATGGGCTTCAACTGGATAGGTAGCACCGGCTTCTGCCGACTACACGAA
GTTGTGGCTTGTGGACTTTGCTAGAGGCCAAGCAGGAAACATGCTTTGCATGTCTTAAACTTTCGACCTGAG
CTCAGGCAAGG

>NOW_sequence_typeA_clone1

ACACACCGCCCGTCGCTCCTACCGATTGGGTGTGCTGGTGAAGTGTTCGGATTGGCGGCTCGGGGCGGTTT
CCGCTCCGGGTTGCTGAGAAGTTCATTAAACCCTCCCACCTAGAGGAAGGAGAAGTCGTAACAAGGTTTCC
GTAGGTGAACCTGCGGAAGGATCATTGAATTATTAAAACCACAATGTGAACCTTATTGTTCCGTGCCTTTGC
TGCCGGCAAGGCAATCAGCTTTGCCTGATTGTACTTGCAAGCTGGTGCGAGTTTTATACTTGCATCAGTGGC
GCTCTGGCATGCTTATACACCAGTGCTAACCACTGTCAAAACCAAACTCTTAAGCTTTGATTGCTATTAATT
GGCAATCTTAACCAAAGACAACTCTCAACAACGGATATCTTGGCTCCCGTATCGATGAAGAACGCAGCGAA
ATGCGATACGTAGTGTGAATTGCAGAATTCCGTGAACCATCGAATCTTTGAACGCATATTGCGCTCGAGTC
CTCGGGCAAGAGCATGTCTGCCTCAGCGTCGGTTTATAACCTCACCCCTCTCTCCTTTTGGAGAGCTGGTTA
GCTTCTAGCTGGCCTTAGGAGTGGATCTGGCTTTCCCATTTGGTTTATTCTGAATGGGTTGGCTGAAGCTTA
GAGGCTTAAGCAAGGACCCGATATGGGCTTCAACTGGATAGGTAGCACCGGCTTCTGCCGACTACACGAAG
TTGTGGCTTGTGGACTTTGCTAGAGGCCAAGCAGGAAACATGCTTTGCATGTCTTAAACTTTCGACCTGAGC
TCAGGCAAGG

>NOW_sequence_typeA_clone2

ACACACCGCCCGTCGCTCCTACCGATTGGGTGTGCTGGTGAAGTGTTCGGATTGGCAGCTTAGGGTGGCAA
CACCTCAGGTCTGCCGAGAAGTTCATTAAACCCTCCCACCTAGAGGAAGGAGAAGTCGTAACAAGGTTTCC
GTAGGTGAACCTGCGGAAGGATCATTGAATTATTAAAACCACAATGTGAACCTTATTGTTCCGTGCCTTTGC
TGCCGGCAAGGCAATCAGCTTTGCCTGATTGTACTTGCAAGCTGGTGCGAGTTTTATACTTGCATCAGTGGC
GCTCTGGCATGCTTATATACCAGTGCTAACCACTGTCAAAACCAAACTCTGAAGCTTTGATTGCTATTAATT
GGCAATCTTAACCAAAGACAACTCTCAACAACGGATATCTTGGCTCTCGCAACGATGAAGAACGCAGCGA
AATGCGATACGTAGTGTGAATTGCAGAATTCCGTGAACCATCGAATCTTTGAACGCATATTGCGCTCGAGC
CCTCGGGCAAGAGCATGTCTGCCTCAGCGTCGGTTTATAACCTCACCCCTCTCTCCTTTTGGAGAGCTGGTT
AGCTTCTAGCTGGCCTTAGGAGTGGATCTGGCTTTCCCATTTGGTTTATTCTGAATGGGTTGGCTGAAGCTT
AGAGGCTTAAGCAAGGACCCGATATGGGCTTCAACTGGATAGGTAGCACCGGCTTCTGCCGACTACACGAA
GTTGTGGCTTGTGGACTTTGCTAGAGGCCAAGCAGGAAACATGCTTTGCATGTCTTAAACTTTCGACCTGAG
CTCAGGCAAGG

>NOW_sequence_typeA_clone3

ACACACCGCCCGTCGCTCCTACCGATTGGGTGTGCTGGTGAAGTGTTCGGATTAGCAGCTTAGGGTGGCAA
CACCTCAGGTCTGCCGAGAAGTTCATTAAACCCTCCCACCTAGAGGAAGGAGAGGTCGTAACAAGGTTTCC
GTAGGTGAACCTGCGGAAGGATCATTGAATTATTAAAACCACAATGTGAACCTTATTGTTCCGTGCCTTTGC
TGCCGGCAAGGCAATCAGCTTTGCCTGATTGTACTTGCAAGCTGGTGCGAGTTTTATACTTGCATCAGTGGC
GCTCTGGCATGCTTATACACCAGTGCTAACCACTGTCAAAACCAAACTCTGAAGCTTTGATTGCTATTAATT
GGCAATCTTAACCAAAGACAACTCTCAACAACGGATATCTTGGCTCTCGCAACGATGAGGAACGCAGCGA
AATGCGATACGTAGTGTGGATTGCAGAATTCCGTGAACCATCGAATCTTTGAACGCATATTGCGCTCGAGC
CCTCGGGCAAGAGCATGTCTGCCTCAGCGTCGGTTTATAACCTCACCCCTCTCTCCTTTTGGAGAGCTGGTT
AGCTTCTAGCTGGCCTTAGGAGTGGATCTGGCTTTCCCATTTGGTTTATTCTGAATGGGTTGGCTGAAGCTT
AGAGGCTTAAGCAAGGACCCGATATGGGCTTCAACTGGATAGGTAGCACCGGCTTCTGCCGACTACACGAA
GTTGTGGCTTGTGGACTTTGCTAGAGGCCAAGCAGGAAACATGCTTTGCATGTCTTAAACTTTCGACCTGAG
CTCAGGCAAGG

>NOW_sequence_typeA_clone4

ACACACCGCCCGTCGCTCCTACCGATTGGGTGTGCTGGTGAAGTGTTCGGATTGGCAGCTTAGGGTGGCAA
CACCTCAGGTCTGCCGAGAAGTTCATTAAACCCTCCCACCTAGAGGAAGGAGAAGTCGTAACAAGGTTTCC
GTAGGTGAACCTGCGGAAGGATCATTGAATTATTAAAACCACAATGTGAACCTTATTGTTCCGTGCCTTTGC
TGCCGGCAAGGCAATCAGCTTTGCCTGATTGTACTTGCAAGCTGGTGCGAGTTTTATACTTGCATCAGTGGC
GCTCTGGCATGCTTATACACCAGTGCTAACCACTGTCAAAACCAAACTCTGAGGCTTTGATTGCTATTAATT
GGCAATCTTAACCAAAGACAACTCTCAACAACGGATATCTTGGCTCTCGCAACGATGAAGAACGCAGCGA
AATGCGATACGTAGTGTGAATTGCAGAATTCCGTGAACCATCGAATCTTTGAACGCATATTGCGCTCGAGC
CCTCGGGCAAGAGCATGTCTGCCTCAGCGTCGGTTTATAACCTCACCCCTCTCTCCTTTTGGAGAGCTGGTT
AGCTTCTAGCTGGCCTTAGGAGTGGATCTGGCTTTCCCATTTGGTTTATTCTGAATGGGTTGGCTGAAGCTT
AGAGGCTTAAGCAAGGACCCGATATGGGCTTCAACTGGATAGGTAGCACCGGCTTCTGCCGACTACACGAA
GTTGTGGCTTGTGGACTTTGCTAGAGGCCAAGCAGGAAACATGCTTTGCATGTCTTAAACTTTCGACCTGAG
CTCAGGCAAGG

>NOW_sequence_typeA_clone5

ACACACCGCCCGTCGCTCCTACCGATTGGGTGTGCTGGTGAAGTGTTCGGATTGGCAGCTTAGGGTGGCAA
CACCTCAGGTCTGCCGAGAAGTTCATTAAACCCTCCCACCTAGAGGAAGGAGAAGTCGTAACAAGGTTTCC
GTAGGTGAACCTGCGGAAGGATCATTGAATTATTAAAACCACAATGTGAACCTTATTGTTCCGTGCCTTTGC
TGCCGGCAAGGCAATCAGCTTTGCCTGATTGTACTTGCAAGCTGGTGCGAGTTTTATACTTGCATCAGTGGC
GCTCTGGCATGCTTATACACCAGTGCTAACCACTGTCAAAACCAAACTCTGAAGCTTTGATTGCTATTAATT
GGCAATCTTAACCAAAGACAACTCTCAACAACGGATATCTTGGCTCTCGCAACGATGAAGAACGCAGCGA
AATGCGATACGTAGTGTGAATTGCAGAATTCCGTGAACCATCGAATCTTTGAACGCATATTGCGCTCGAGC
CCTCGGGCAAGAGCATGTCTGCCTCGGCGTCGGTTTATAACCTCACCCCTCTCTCCTTTTGGAGAGCTGGTT
AGCTTCTAGCTGGCCTTAGGAGTGGATCTGGCTTTCCCATTTGGTTTATTCTGAATGGGTTGGCTGAAGCTT
AGAGGCCTAAGCAAGGACCCGATATGGGCTTCAACTGGATAGGTAGCACCGGCTTCTGCCGACTACACGA
AGTTGTGGCTTGTGGACTTTGCTAGAGGCCAAGCAGGAAACATGCTTTGCATGTCTTAAACTTTCGACCTGA
GCTCAGGCAAGG

>NOW_sequence_typeA_clone6

ACACACCGCCCGTCGCTCCTACCGATTGGGTGTGCTGGTGAAGTGTTCGGATTGGCAGCTTAGGGTGGCAA
CACCTCAGGTCTGCCGAGAAGTTCATTAAACCCTCCCACCTAGAGGAAGGAGAAGTCGTAACAAGGTTTCC
GTAGGTGAACCTGCGGAAGGATCATTGAATTATTAAAACCACAATGTGAACCTTATTGTTCCGTGCCTTTGC
TGCCGGCAAGGCAATCAGCTTTGCCTGATTGTACTTGCAAGCTGGTGCGAGTTTTATACTTGCATCAGTGGC
GCTCTGGCATGCTTATACACCAGTGCTAACCACTGTCAAAACCAAACTCTGAAGCTTTGATTGCTATTAATT
GGCAATCTTAACCAAAGACAACTCTCAACAACGGATATCTTGGCTCTCGCAACGATGAAGAACGCAGCGA
AATGCGATACGTAGTGTGAATTGCAGAATTCCGTGAACCATCGAATCTTTGAACGCATATTGCGCTCGAGC
CCTCGGGCAAGAGCATGTCTGCCTCGGCGTCGGTTTATAACCTCACCCCTCTCTCCTTTTGGAGAGCTGGTT

AGCTTCTAGCTGGCCTTAGGAGTGGATCTGGCTTTCCCATTTGGTTTATTCTGAATGGGTTGGCTGAAGCTT
AGAGGCCTAAGCAAGGACCCGATATGGGCTTCAACTGGATAGGTAGCACCGGCTTCTGCCGACTACACGA
AGTTGTGGCTTGTGGACTTTGCTAGAGGCCAAGCAGGAAACATGCTTTGCATGTCTTAAACTTTCGACCTGA
GCTCAGGCAAGG

>NOW_sequence_typeA_clone7

ACACACCGCCCGTCGCTCCTACCGATTGGGTGTGCTGGTGAAGTGTTCGGATTGGCAGCTTAGGGTGGCAA
CACCTCAGGTCTGCCGAGAAGTTCATTAAACCCTCCCACCTAGAGGAAGGAGAAGTCGTAACAAGGTTTCC
GTAGGTGAACCTGCGGAAGGATCATTGAATTATTAAAACCACAATGTGAACCTTATTGTTCCGTGCCTTTGC
TGCCGGCAAGGCAATCAGCTTTGCCTGATTGTACTTGCAAGCTGGTGCGAGTTTTATACTTGCATCAGTGGC
GCTCTGGCATGCTTATACACCAGCGCTAACCACTGTCAAAACCAAACTCTGAAGCTTTGATTGCTATTAATT
GGCAATCTTAACCAAAGACAACTCTCAACAACGGATATCTTGGCTCTCGCAACGATGAAGAACGCAGCGA
AATGCGATACGTAGTGTGAATTGCAGAATTCCGTGAACCATCGAATCTTTGAACGCATATTGCGCTCGAGC
CCTCGGGCAAGAGCATGTCTGCCTCAGCGTCGGTTTATAACCTCACCCCTCTCTCCTTTTGGAGAGCTGGTT
AGCTTCTAGCTGGCCTTAGGAGTGGATCTGGCTTTCCCATTTGGTTTATTCTGAATGGGTTGGCTGAAGCTT
AGAGGCTTAAGCAAGGACCCGATATGGGCTTCAACTGGATAGGTAGCACCGGCTTCTGCCGACTACACGAA
GTTGTGGCTTGTGGACTTTGCTAGAGGCCAAGCAGGAAACATGCTTTGCATGTCTTAAACTTTCGACCTGAG
CTCAGGCAAGG

>NOW_sequence_typeA_clone9

ACACACCGCCCGTCGCTCCTATCGATTGGGTGTGCTGGTGAAGTGTTCGGATTGGCAGCTTAGGGTGGCAA
CACCTCAGGTCTGCCGAGAAGTTCATTAAACCCTCCCACCTAGAGGAAGGAGAAGTCGTAACAAGGTTTCC
GTAGGTGAACCTGCGGAAGGATCATTGAATTATTAAAACCACAATGTGAACCTTATTGTTCCGTGCCTTTGC
TGCCGGCAAGGCAATCAGCTTTGCCTGATTGTACCTGCAAGCTGGTGCGAGTTTTATACTTGCATCAGTGGC
GCTCTGGCATGCTTATACACCAGTGCTAACCACTGTCAAAACCAAACTCTGAAGCTTTGATTGCTATTAATT
GGCAATCTTAACCAAAGACAACTCTCAACAACGGATATCTTGGCTCTCGCAACGATGAAGAACGCAGCGA
AATGCGATACGTAGTGTGAATTGCAGAATTCCGTGAACCATCGAATCTTTGAACGCATATTGCGCTCGAGC
CCTCGGGCAAGAGCATGACTGCCTCAGCGTCGGTTTATAACCTCACCCCTCTCTCCTTTTGGAGAGCTGGTT
AGCTTCTAGCTGGCCTTAGAAGTGGATCTGGCTTTCCCATTTGGTTTATTCTGAATGGGTTGGCTGAAGCTT
AGAGGCTTAAGCAAGGACCCGATATGGGCTTCAACTGGATAGGTAGCACCGGCTTCTGCCGACTACACGAA
GTTGTGGCTTGTGGACTTTGCTAGAGGCCAAGCAGGAAACATGCTTTGCATGTCTTAAACTTTCGACCTGAG
CTCAGGCAAGG

>NOW_sequence_typeA_clone10

ACACACCGCCCGTCGCTCCTACCGATTGGGTGTGCTGGTGAAGTGTTCGGATTGGCAGCTTAGGGTGGCAA
CACCTCAGGTCTGCCGAGAAGTTCATTAAACCCTCCCACCTAGAGGAAGGAGAAGTCGTAACAAGGTTTCC
GTAGGTGAACCTGCGGAAGGATCATTGAATCATTAAAACCACAATGTGAACCTTATTGTTCCGTGCCTTTGC
TGCCGGCAAGGCAATCAGCTTTGCCTGATTGTACTTGCAAGCTGGTGCGAGTTTTATACTTGCATCAGTGGC
GCTCTGGCATGCTTATACACCAGTGCTAACCACTGTCAAAACCAAACTCTGAAGCTTTGATTGCTATTAATT
GGCAATCTTAACCAAAGACAACTCTCAACAACGGATATCTTGGCTCTCGCAACGATGAAGAACGCAGCGA
AATGCGATACGTAGTGTGAATTGCAGAATTCCGTGAACCATCGAATCTTTGAACGCATATTGCGCTCGAGC
CCTCGGGCAAGAGCATGTCTGCCTCAGCGTCGGTTTATACCCTCACCCCTCTCTCCTTTTGGAGGGCTGGTC
AGCTTCTAGTTGGCCTCAGGGGTGGATCTGGCTTTCCCAATTGGTTCACTCCGATTGGGTTGGCTGAAGCTT
AGAGGCTTAAGCAAGGACCCGACATGGGCTTCAACTGGATAGGTAGCACCGGCTTCTGCCGACTACACGA
AGTTGTGGCTTGTGGACTTTGCTAGAGGCCAAGCAGGAAACATGCTTTGCATGTCTTAAACTTTCGACCTGA
GCTCAGGCAAGG

>NOW_sequence_typeA_clone11

ACACACCGCCCGTCGCTCCTACCGATTGGGTGTGCTGGTGAAGTGTTCGGATTGGCAGCTTAGAGTGGCAA
CACCTCAGGTCTGCCGAGAAGTTCATTAAACCCTCCCACCTAGAGGAAGGAGAAGTCGTAACAAGGTTTCC
GTAGGTGAACCTGCGGAAGGATCATTGAATTATTAAAACCACAATGTGAACCTCATTGTTCCGTGCCTTTGC
TGCCGGCAAGGCAATCAGCTTTGCCTGATTGTACTTGCAAGCTGGTGCGAGTTTTATACTTGCATCAGTGGC
GCTCTGGCATGCTTATGCACCAGTGCTAACCACTGTCAAAACCAAACTCTGAAGCTTTGATTGCTATTAATT

GGCAATCTTAACCAAAGACAACTCTCAACAACGGATATCTTGGCTCTCGCAACGATGAAGAACGCAGCGA
AATGCGATACGTAGTGTGAATTGCAGAATTCCGTGAACCATCGAATCTTTGAACGCATATTGCGCTCGAGC
CCTCGGGCAAGAGCATGTCTGCCTCAGCGTCGGTTTATAACCTCACCCCTCTCTCCTTTTGGAGAGCTGGTT
AGCTTCTAGCTGGCCTTAGGAGTGGATCTGGCTTTCCCATTTGGTTTATTCTGAATGGGTTGGCTGAAGCTT
AGAGGCTTAAGCAAGGGCCCGATATGGGCTTCAACTGGATAGGTAGCACCGGCTTCTGCCGACTACACGAA
GTTGTGGCTTGTGGACTTTGCTAGAGGCCAAGCAGGAAACATGCTTTGCATGTCTTAAACTTTCGACCTGAG
CTCAGGCAAGG

>NOW_sequence_typeA_clone12

ACACACCGCCCGTCGCTCCTACCGATTGGGTGTGCTGGTGAAGTGTTCGGATTGGCAGCTTAGGGTGGCAA
CACCTCAGGTCTGCCGAGAAGTTCATTAAACCCTCCCACCTAGAGGAAGGAGAAGTCGTAACAAGGTTTCC
GTAGGTGAACCTGCGGAAGGATCATTGAATTATTAAAACCACAATGTGAACCTTATCGTTCCGTGCCTTTGC
TGCCGGCAAGGCAATCAGCTTTGCCTGATTGTACTTGCAAGCTGGTGCGAGTTTTATACTTGCATCAGTGGC
GCTCTGGCATGCTTATACACCAGTGCTAACCACTGTCAAAACCAAACTCTGAAGCTTTGATTGCTATTAATT
GGCAATCTTAACCAAAGACAACTCTCAACAACGGATATCTTGGCTCTCGCAACGATGAAGAACGCAGCGA
AATGCGATACGTAGTGTGAATTGCAGAATTCCGTGAACCATCGAATCTTTGAACGCATATTGCGCTCGAGC
CCTCGGGCAAGAGCATGTCTGCCTCAGCGTCGGTTTATAACCTCACCCCTCTCTCCTTTTGGAGAGCTGGTT
AGCTTCTAGCTGGCCTTAGGGGTGGATCTGGCTTTCCCATTTGGTTTATTCTGAATGGGTTGGCTGAAGCTT
AGAGGCTTAAGCAAGGACCCGATATGGGCTTCAACTGGATAGGTAGCACCGGCTTCTGCCGACTACACGAA
GTTGTGGCTTGTGGACTTTGCTAGAGGCCAAGCAGGAAACATGCTTTGCATGTTTTAAACTTTCGACCTGAG
CTCAGGCAAGA

>NOW_sequence_typeA_clone13

ACACACCGCCCGTCGCTCCTACCGATTGGGCGTGCTGGTGAAGTGTTCGGATTGGCAGCTTAGGGTGGCAA
CACCTCAGGTCTGCCGAGAAGTTCATTAAACCCTCCCACCTAGAGGAAGGAGAAGTCGTAACAAGGTTTCC
GTAGGTGAACCTGCGGAAGGATCATTGAATTATTAAAACCACAATGTGAACCTTATTGTTCCGTGCCTTTGC
TGCCGGCAAGGCAATCAGCTTTGCCTGATTGTACTTGCAAGCTGGTGCGAGTTTTATACTTGCATCAGTGGC
GCTCTGGCATGCTTATACACCAGTGCTAACCACTGTCAAAACCAAACTCTGAAGCTTTGATTGCTATTAATT
GGCAATCTTAACCAAAGACAACTCTCAACAACGGATATCTTGGCTCTCGCAACGATGAAGAACGCAGCGA
AATGCGATACGTAGTGTGAATTGCAGAATTCCGTGAACCATCGAATCTTTGAACGCATATTGCGCTCGAGC
CCTCGGGCAAGAGCATGTCTGCCTCAGCGTCGGTTTATACCCTCACCCCTCTCTCCTTTTGGAGGGCTGGTC
AGCTTCTAGTTGGCCTCAGGGGTGGATCTGGCTTTCCCAATTGGTTCACTCCGATTGGGTTGGCTGAAGCTT
AGAGGCTTAAGCAAGGACCCGATATGGGCTTCAACTGGATAGGTAGCACCGGCTTCTGCCGACTACACGAA
GTTGTGGCTTGTGGACTTTGCTAGAGGCCAAGCAGGAAACATGCTTTGCATGTCTTAAACTTTCGACCTGAG
CTCAGGCAGGG

>NOW_sequence_typeA_clone14

ACACACCGCCCGTCGCTCCTACCGATTGGGTGTGCTGGTGAAGTGTTCGGATTGGCAGCTTAGGGTGGCAA
CACCTCAGGTCTGCCGAGAAGTTCATTAAACCCTCCCACCTAGAGGAAGGAGAAGTCGTAACAAGGTTTCC
GTAGGTGAACCTGCGGAAGGATCATTGAATTATTAAAACCACAATGTGAACCTTATTGTTCCGTGCCTTTGC
TGCCGGCAAGGCAATCAGCTTTGCCTGATTGTACTTGCAAGCTGGTGCGAGTTTTATACTTGCATCAGTGGC
GCTCTGGCATGCTTATACACCAGTGCTAACCACTGTCAAAACCAAACTCTGAAGCTTTGATTGCTATTAATT
GGCAATCTTAACCAAAGACAACTCTCAACAACGGATATCTTGGCTCTCGCAACGATGAAGAACGCAGCGA
AATGCGATACGTAGTGTGAATTGCAGAATTCCGTGAACCATCGAATCTTTGAACGCATATTGCGCTCGAGC
CCTCGGGCAAGAGCATGTCTGCCTCAGCGTCGGTTTATAACCTCACCCCTCTCTCCTTTTGGAGAGCTGGTT
AGCTTCTAGCTGGCCTTAGGAGTGGATCTGGCTTTCCCATTTGGTTTATTCTGAATGGGTTGGCTGAAGCTT
AGAGGCTTAAGCAAGGACCCGATATGGGCTTCAACTGGATAGGTAGCACCGGCTTCTGCCGACTACACGAA
GTTGTGGCTTGTGGACTTTGCTAGAGGCCAAGCAGGAAACATGCTTTGCATGTCTTAAACTTTCGACCTGAG
CTCAGGCAAGG

>NOW_sequence_typeA_clone15

ACACACCGCCCGTCGCTCCTACCGATTGGGTGTGCTGGTGAAGTGTTCGGATTGGCAGCTTAGGGTGGCAA
CACCTCAGGTCTGCCGAGAAGTTCATTAAACCCTCCCACCCAGAGGAAGGAGAAGTCGTAACAAGGTTTCC
GTAGGTGAACCTGCGGAAGGATCATTGAATTATTAAAACCACAATGTGAACCTTATTGTTCCGTGCCTTTGC
TGCCGGCAAGGCAATCAGCTTCGCCTGATTGTACTTGCAAGCTGGTGCGAGTTTTATACTTGCATCAGTGGC
GCTCTGGCATGCTTATACACCAGTGCTAACCACTGTCAAAACCAAACTCTGAAGCTTTGATTGCTATTAATT
GGCAATCTTAACCAAAGACAACTCTCAACAACGGATATCTTGGCTCTCGCAACGATGAAGAACGCAGCGA
AATGCGATACGTAGTGTGAATTGCAGAATTCCGTGAACCATCGAATCTTTGAACGCATATTGCGCTCGAGC
CCTCGGGCAAGAGCATGTCTGCCTCAGCGTCGGTTTATAACCTCACCCCTCTCTCCTTTTGGAGAGCTGGTT
AGCTTCTAGCTGGCCTTAGGAGTGGATCTGGCTTTCCCATTTGGTTTATTCTGAATGGGTTGGCTGAAGCTT
AGAGGCTTAAGCAAGGACCCGATATGGGCTTCAACTGGATAGGTAGCACCGGCTTCTGCCGACTACACGAA
GTTGTGGCTTGTGGACTTTGCTAGAGGCCAAGCAGGAAACATGCTTTGCATGTCTTAAACTTTCGACCTGAG
CTCAGGCAAGG

>NOW_sequence_typeA_clone16

ACACACCGCCCGTCGCTCCTACCGATTGGGTGTGCTGGTGAAGTGTTCGGATTGGCAGCTTAGGGTGGCAA
CACCTCAGGTCTGCCGAGAAGTTCATTAAACCCTCCCACCTAGAGGAAGGAGAAGTCGTAACAAGGTTTCC
GTAGGTGAACCTGCGGAAGGATCATTGAATTATTAAAACCACAATGTGAACCTTATTGTTCCGTGCCTTTGC
TGCCGGCAAGGCAATCAGCTTTGCCTGATTGTACTTGCAAGCTGGTGCGAGTTTTATACTTGCATCAGTGGC
GCTCTGGCATGCTTATACACCAGTGCTAACCACTGTCAAAACCAAACTCTGAAGCTTTGATTGCTATTAATT
GGCAATCTTAACCAAAGACAACTCTCAACAACGGATATCTTGGCTCTCGCAACGATGAAGAACGCAGCGA
AATGCGATACGTAGTGTGAATTGCAGAATTCCGTGAACCATCGAATCTTTGAACGCATATTGCGCTCGAGC
CCTCGGGCAAGAGCATGTCTGCCTCAGCGTCGGTTTATAACCTCACCCCTCTCTCCTTTTGGAGAGCTGGTT
AGCTTCTAGCTGGCCTTAGGAGTGGATCTGGCTTTCCCATTTGGTTTATTCTGAATGGGTTGGCTGAAGCTT
AGAGGCTTAAGCAAGGACCCGATATGGGCTTCAACTGGATAGGTAGCACCGGCTTCTGCCGACTACACGAA
GTTGTGGCTTGTGGACTTTGCTAGAGGCCAAGCAGGAAACATGCTTTGCATGTCTTAAACTTTCGACCTGAG
CTCAGGCAAGG

>NOW_sequence_typeB

ACACACCGCCCGTCGCTCCTACCGATTGGGTGTGCTGGTGAAGTGTTCGGATTGGCAGCTTAGGGTGGCAA
CACCTCAGGTCTGCCGAGAAGTTCATTAAACCCTCCCACCTAGAGGAAGGAGAAGTCGTAACAAGGTTTCC
GTAGGTGAACCTGCGGAAGGATCATTGAATCGATCGAATCCACACCGGTAACCAAACGTCGCCCCCCTGTG
GTGCATTCTCCGGATCTCCGGCGTTTCACCCTGGGCGTCGGCCCCTGGGCTGGGGCTCTCACGAGCCGCTTT
TCAGGTCCGACGGGCGCCTCCCTTGGGCTCACCCCCTGGGGCTGGCGTCGGCCAAAACCCCTGTATCCAAC
CTTTTTTTAACACACCCCAAACCACAACCAACTCTGAAGCATCTTTGGTGGCCCGGCCTCGTGCCGTCCACT
CCAAACCAAAGACGACTCTCAACAACGGATATCTTGGCTCCCGTATCGATGAAGAACGCAGCGAAATGCG
ATACGTAGTGTGAATTGCAGAATTCCGTGAACCATCGAATCTTTGAACGCAAATTGCGCCCGAGACTTCGG
CCGAGGGCATGTCTGCCTCAGCGTCGGTTTACACCCTCGCCCTCCCCCACCCTGTGTGGTGGGGTGTTGGTG
CGGATCTGGCCCTCCCGGCTCCGCTCTGTTGAGCGCCCCGGGTTGGCTGAAGCCCAGAGACTTGAGCATGG
ACCCCGTTTGTAGGGCAATGGCTTGGTAGGTAGGCACCCCCTACGCAGCCTGCCGTTGCCCGAGGGGACTT
TGCTGGAGGCCCAGCAGGAATCCAGCTGTTTCGGCAGCCGGACTACTCACTTATTCGACCTGAGCTCAGGC
AAGA
